# microRNA-92a promotes CNS autoimmunity by modulating the regulatory and inflammatory T cell balance

**DOI:** 10.1172/JCI155693

**Published:** 2022-05-16

**Authors:** Mai Fujiwara, Radhika Raheja, Lucien P. Garo, Amrendra K. Ajay, Ryoko Kadowaki-Saga, Sukrut H. Karandikar, Galina Gabriely, Rajesh Krishnan, Vanessa Beynon, Anu Paul, Amee Patel, Shrishti Saxena, Dan Hu, Brian C. Healy, Tanuja Chitnis, Roopali Gandhi, Howard L. Weiner, Gopal Murugaiyan

**Affiliations:** 1 Ann Romney Center for Neurologic Diseases, Brigham and Women’s Hospital and Harvard Medical School, Boston, Massachusetts, USA.; 2 Pulmonary Center, Department of Medicine, Boston University School of Medicine, Boston, Massachusetts, USA.; 3 Renal Division, Department of Medicine, Brigham and Women’s Hospital and Harvard Medical School, Boston, Massachusetts, USA.

**Keywords:** Autoimmunity, Inflammation, Autoimmune diseases, Multiple sclerosis, T cells

## Abstract

A disequilibrium between immunosuppressive Tregs and inflammatory IL-17–producing Th17 cells is a hallmark of autoimmune diseases, including multiple sclerosis (MS). However, the molecular mechanisms underlying the Treg and Th17 imbalance in CNS autoimmunity remain largely unclear. Identifying the factors that drive this imbalance is of high clinical interest. Here, we report a major disease-promoting role for microRNA-92a (miR-92a) in CNS autoimmunity. miR-92a was elevated in experimental autoimmune encephalomyelitis (EAE), and its loss attenuated EAE. Mechanistically, miR-92a mediated EAE susceptibility in a T cell–intrinsic manner by restricting Treg induction and suppressive capacity, while supporting Th17 responses, by directly repressing the transcription factor Foxo1. Although miR-92a did not directly alter Th1 differentiation, it appeared to indirectly promote Th1 cells by inhibiting Treg responses. Correspondingly, miR-92a inhibitor therapy ameliorated EAE by concomitantly boosting Treg responses and dampening inflammatory T cell responses. Analogous to our findings in mice, miR-92a was elevated in CD4^^^+^^^ T cells from patients with MS, and miR-92a silencing in patients’ T cells promoted Treg development but limited Th17 differentiation. Together, our results demonstrate that miR-92a drives CNS autoimmunity by sustaining the Treg/Th17 imbalance and implicate miR-92a as a potential therapeutic target for MS.

## Graphical abstract

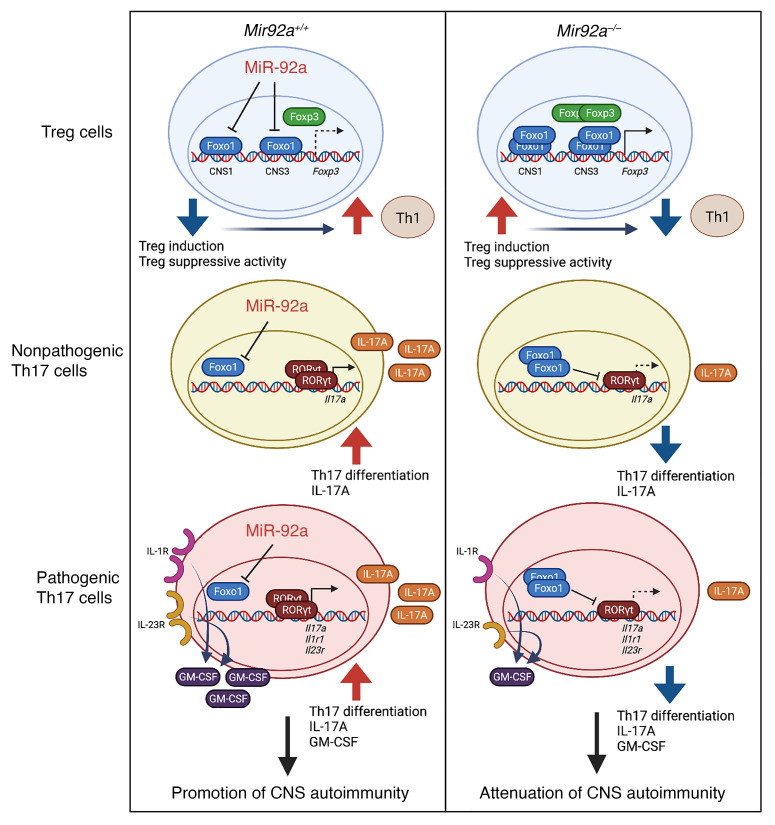

## Introduction

Multiple sclerosis (MS) and its animal model of experimental autoimmune encephalomyelitis (EAE) are mediated by dysregulated autoreactive T cell responses in the CNS that perpetuate neuroinflammation and neuronal damage (1). The differentiation of naive CD4^+^ T cells into immunosuppressive Tregs versus inflammatory Th cells, such as Th1 and Th17 cells, is tightly regulated by complex networks of cytokines and transcription factors that initiate these lineage-specific transcriptional programs (2). However, in the context of MS and other autoimmune diseases, the expansion of pathogenic effector Th cells can outweigh the suppressive capacity of Tregs, leaving the tissue inflammation unchecked (2, 3). Thus, a better understanding of the molecular mechanisms that drive the imbalance between Tregs and inflammatory Th cells would critically aid the development of therapeutics for MS and other autoimmune diseases.

Tregs are a subset of CD4^+^ T cells that play a prominent role in the maintenance of immunological self-tolerance and prevention of autoimmunity in mice and humans (2). The commitment of naive CD4^+^ T cells toward the Treg lineage requires the cytokine TGF-β, which induces the expression of Foxp3 — a key transcription factor that is indispensable for maintenance of the Treg phenotype and suppressive function (4). Loss-of-function mutations in the *FOXP3* gene lead to a severe systemic autoimmune disease called immune dysregulation polyendocrinopathy enteropathy X-linked (IPEX) syndrome in humans and to fatal multiorgan autoimmunity in mice (2). Impairments in Treg frequencies and suppressive function are associated with EAE and MS (5–8) and are thought to be caused by various factors, including elevated levels of inflammatory cytokines. Inflammatory signals can not only interfere with the induction of Tregs, but can also promote preexisting Tregs to acquire an effector-like inflammatory phenotype with diminished suppressive function (2, 8–10).

In contrast to Tregs, IL-17–producing Th17 cells are known to promote several autoimmune diseases, including EAE and MS (11, 12). Cytokines such as IL-17A, secreted by Th17 cells, are detected in the CNS of EAE mice and active lesions of patients with MS (12, 13). The proinflammatory cytokine IL-6, together with TGF-β, induces expression of the Th17-specific transcription factor retinoic acid–related orphan receptor γ t (RORγt) to initiate Th17 differentiation. This gives rise to IL-17A–secreting Th17 cells (14–16) that cannot effectively promote autoimmune inflammation and have therefore been characterized as “nonpathogenic” (17, 18). When these cells are further stimulated with additional cytokines such as IL-23 and/or IL-1β secreted by antigen-presenting cells (APCs), they can differentiate into “pathogenic” Th17 cells capable of mediating autoimmune inflammation in vivo (19, 20). One key feature required for pathogenic Th17 differentiation is the upregulation of the IL-23 receptor (IL-23R) and IL-1 receptor (IL-1R). Signaling of both IL-23R and IL-1R is critical for inducing GM-CSF, which confers the ability of these cells to drive CNS inflammation during EAE (21, 22). In addition to Th17 cells, IFN-γ–secreting inflammatory Th1 cells also play a major role in perpetuating tissue inflammation and CNS autoimmunity (11). Increased levels of IFN-γ– and Th1-promoting cytokines, including IL-12, have been observed in EAE and MS (23, 24).

Recent work by us and others has revealed that microRNAs (miRNAs) are major regulators of immune responses in a variety of disease contexts (25), including CNS autoimmunity in mice (26–28) and humans (29). miRNAs are a class of short, noncoding RNAs that are indispensable for regulating gene expression by binding the 3′-UTR of target mRNAs to enhance mRNA degradation or inhibit mRNA translation (30). In patients with MS, miRNA expression profiles have been found to differ significantly within CNS lesions, immune cell subsets, circulating plasma and sera, and cerebrospinal fluid (CSF), as compared with healthy controls (HCs) (31, 32). However, specific miRNA pathways that directly connect clinical disease activity with pathogenic and regulatory immune mechanisms in EAE and MS, including the Treg/Th17 imbalance, have not been well defined. To identify miRNAs that are clinically relevant in MS, we previously profiled more than 600 miRNAs in sera from patients with MS and from HCs (33). microRNA-92a (miR-92a) was identified as one of the most significantly increased miRNAs in patients with MS (33). Intriguingly, miR-92a levels were found to positively correlate with the extended disability standard scale (EDSS) (33), an MS clinical parameter that measures disability progression (34). Another study also showed significant positive correlations between miR-92a levels in the sera from patients with MS and MRI-based measurements of MS lesion volumes (brain T1/T2 ratio; ref. 35). These findings suggest an important link between miR-92a and MS pathogenesis (33, 35)and led us to investigate the functional role of miR-92a in CNS autoimmunity.

Here, we report a major disease-promoting role for miR-92a in EAE and MS. miR-92a is elevated in EAE, and its loss attenuates disease severity in EAE. By targeting the transcription factor Foxo1, miR-92a promoted Th17 differentiation, while impairing the differentiation and suppressive function of Tregs, thereby promoting CNS autoimmunity. Although miR-92a did not directly alter Th1 differentiation, it appeared to indirectly promote Th1 cells by inhibiting Treg responses to further sustain CNS autoimmunity. Correspondingly, miR-92a silencing effectively ameliorated EAE by reversing the Treg/Th17 imbalance. Finally, we show that miR-92a was elevated in CD4^+^ T cells from patients with MS and that silencing miR-92a could promote Treg induction while impairing Th17 differentiation in both CD4^+^ T cells from both HCs and patients with MS. miR-92a constitutes a powerful target that modulates multiple pathways downstream of Foxo1, a key target of this miRNA to reinstate the equilibrium between regulatory and inflammatory T cells in EAE and MS. Our results provide functional insights into mechanisms of CNS autoimmunity mediated by miR-92a and implicate miR-92a silencing as a potential therapeutic approach that could modulate the Treg/Th17 imbalance in MS.

## Results

### miR-92a is upregulated in EAE and its loss attenuates EAE.

**Figure 1:**
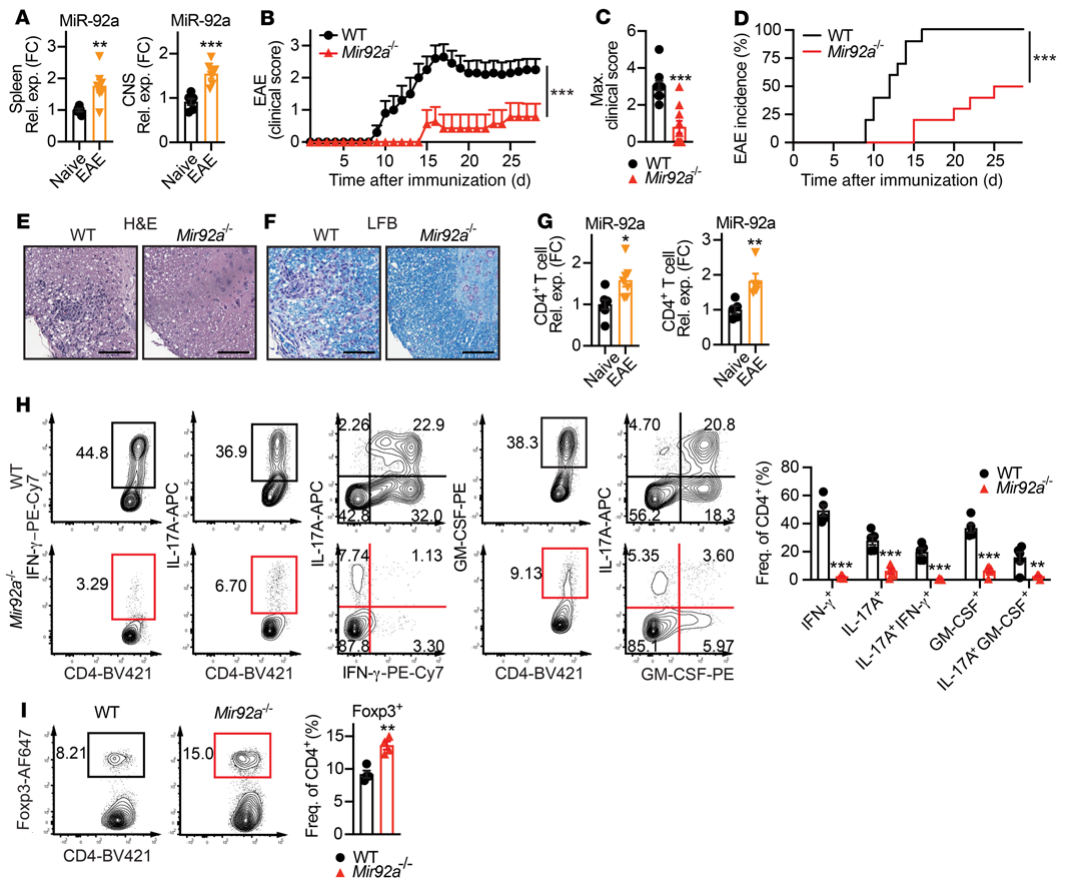
*Mir92a^–/–^* mice develop attenuated EAE. (**A**) qPCR analysis of miR-92a in total splenocytes (spleen, left) and spinal cords (CNS, right) from WT mice at EAE onset (*n =* 6–7). (**B**) Clinical EAE scores for WT and *Mir92a^–/–^* mice (*n =* 9–10) via standardized EAE clinical scale for ascending paralysis from 0–5 on each day during the observation period for each mouse. (**C**) Maximum (Max.) EAE clinical scores reached for each individual WT and *Mir92a^–/–^* mouse (*n =* 9–10) during the observation period from **B**. (**D**) Percentage of disease incidence for WT and *Mir92a^–/–^* mice (*n =* 9–10) on each day during the observation period from **B**. (**E** and **F**) Representative histopathological sections of spinal cords from WT and *Mir92a^–/–^* mice at peak EAE, showing immune cell infiltration via H&E staining (**E**) and demyelination via LFB staining (**F**). Scale bars: 100 μm. Original magnification, ×100. (**G**) qPCR analyses of miR-92a in splenic CD4^+^ T cells from naive WT mice versus that in EAE WT mice (*n =* 7) (left) and miR-92a in splenic CD4^+^ T cells from naive WT mice versus CNS-infiltrating CD4^+^ T cells from EAE WT mice (*n =* 5) (right). FC, fold change. (**H**) Representative flow cytometric plots (left) and frequencies (right) of IFN-γ^+^, IL-17A^+^, IFN-γ^+^IL-17A^+^, GM-CSF^+^, and IL-17A^+^GM-CSF^+^CD4^+^ T cells in the spleens of WT and *Mir92a^–/–^* mice at peak EAE (*n =* 5–6). PE-Cy7, phycoerythrin/cyanine7. (**I**) Representative flow cytometric plots and frequencies of Foxp3^+^ Tregs in the spleens of WT and *Mir92a^–/–^* mice at EAE onset (*n =* 4). AF647, Alexa Fluor 647. Data are representative of 2 or more independent experiments and indicate the mean ± SEM. *P < 0.05, **P < 0.01, and ***P < 0.001, by unpaired, 2-tailed Student’s *t* test (**A**, **C**, **G**, and **I**), Mann-Whitney *U* test (**B**), log-rank (Mantel-Cox) test (**D**), or 1-way ANOVA with Šidák’s multiple-comparison test between WT and *Mir92a^–/–^* mice within each condition (**H**). Rel. exp., relative expression; Freq., frequency; BV421, Brilliant Violet 421.

To determine whether miR-92a may play a key role in CNS autoimmunity, we first examined miR-92a levels in naive WT mice and WT mice with EAE. Increased miR-92a expression in splenocytes and spinal cords (CNS) during EAE (Figure 1A) hinted at the involvement of miR-92a in this context. For a close evaluation of miR-92a function in EAE, we next used miR-92a–deficient (*Mir92a^–/–^*) mice. We observed that naive *Mir92a^–/–^* mice were viable, fertile, and lacked any apparent gross abnormalities, as previously reported (36). We also performed an analysis of thymic and peripheral myeloid and lymphoid populations in these mice. These analyses revealed no overt defects in the sizes of spleens or lymph nodes (LNs) or in the frequencies of splenic DCs, macrophages, B cells, and NK cells (Supplemental Figure 1, A–E; supplemental material available online with this article; https://doi.org/10.1172/JCI155693DS1). In addition, we found largely unaltered frequencies of thymocytes, including both CD4^+^ and CD8^+^ single-positive and double-positive populations, as well as Foxp3^+^ Tregs in both the periphery and the thymus (Supplemental Figure 1, F–I). However, when we induced EAE in *Mir92a^–/–^* mice, we found that miR-92a loss strikingly delayed EAE onset and attenuated disease severity (Figure 1, B and C). This EAE attenuation was characterized by reduced disease incidence (Figure 1D), with minimal inflammation and demyelination in the spinal cord (Figure 1, E and F).

Since CD4^+^ T cells are indispensable for EAE pathogenesis (11), we next examined miR-92a levels within CD4^+^ T cells between WT naive and EAE mice. We found that miR-92a expression was upregulated in FACS-sorted CD4^+^ T cells (Supplemental Figure 1J) from both the spleen and CNS during EAE compared with its expression in naive WT mice (Figure 1G). We next assessed whether CD4^+^ Th cell responses were altered in *Mir92a^–/–^* mice during EAE. Intriguingly, we observed reduced frequencies of IFN-γ^+^ Th1 cells, IL-17A^+^ Th17 cells, and IFN-γ^+^IL-17A^+^ double producers in *Mir92a^–/–^* mice at EAE onset (Supplemental Figure 2A) and during peak disease (Figure 1H). GM-CSF is a key pathogenic cytokine primarily secreted by encephalitogenic CD4^+^ T cells, including Th17 cells, during EAE (21, 22). Consistent with attenuated EAE, we also found reduced GM-CSF within total CD4^+^ T cells, including specifically within Th17 cells, in *Mir92a^–/–^* mice (Supplemental Figure 2A and Figure 1H). We also observed decreased frequencies of inflammatory Th1 and Th17 cells in splenocytes from *Mir92a^–/–^* mice restimulated with myelin oligodendrocyte glycoprotein 35–55 (MOG_35–55_) peptide in vitro in a recall assay (Supplemental Figure 2B). In stark contrast to reduced inflammatory responses, we found increased frequencies of Foxp3^+^ Tregs in *Mir92a^–/–^* mice (Figure 1I). Of note, we saw no differences in IFN-γ, IL-17A, GM-CSF, or Foxp3 expression in peripheral CD4^+^ T cells between *Mir92a^–/–^* and WT mice that were immunized only with CFA containing desiccated *Mycobacterium tuberculosis* (CFA/Mtb) (Supplemental Figure 3, A and B), suggesting that the altered CD4^+^ T cell responses observed in *Mir92a^–/–^* mice were not due to general defects in global adjuvant-triggered inflammation during EAE, but rather to impaired CNS autoimmunity. Together, these results suggested that miR-92a loss resulted in attenuated EAE associated with reduced Th1 and Th17 cells and increased Tregs and implicated a potential role for miR-92a in Th cell responses during EAE.

### T cell–intrinsic miR-92a drives EAE by differentially regulating Tregs and Th17 cells.

**Figure 2:**
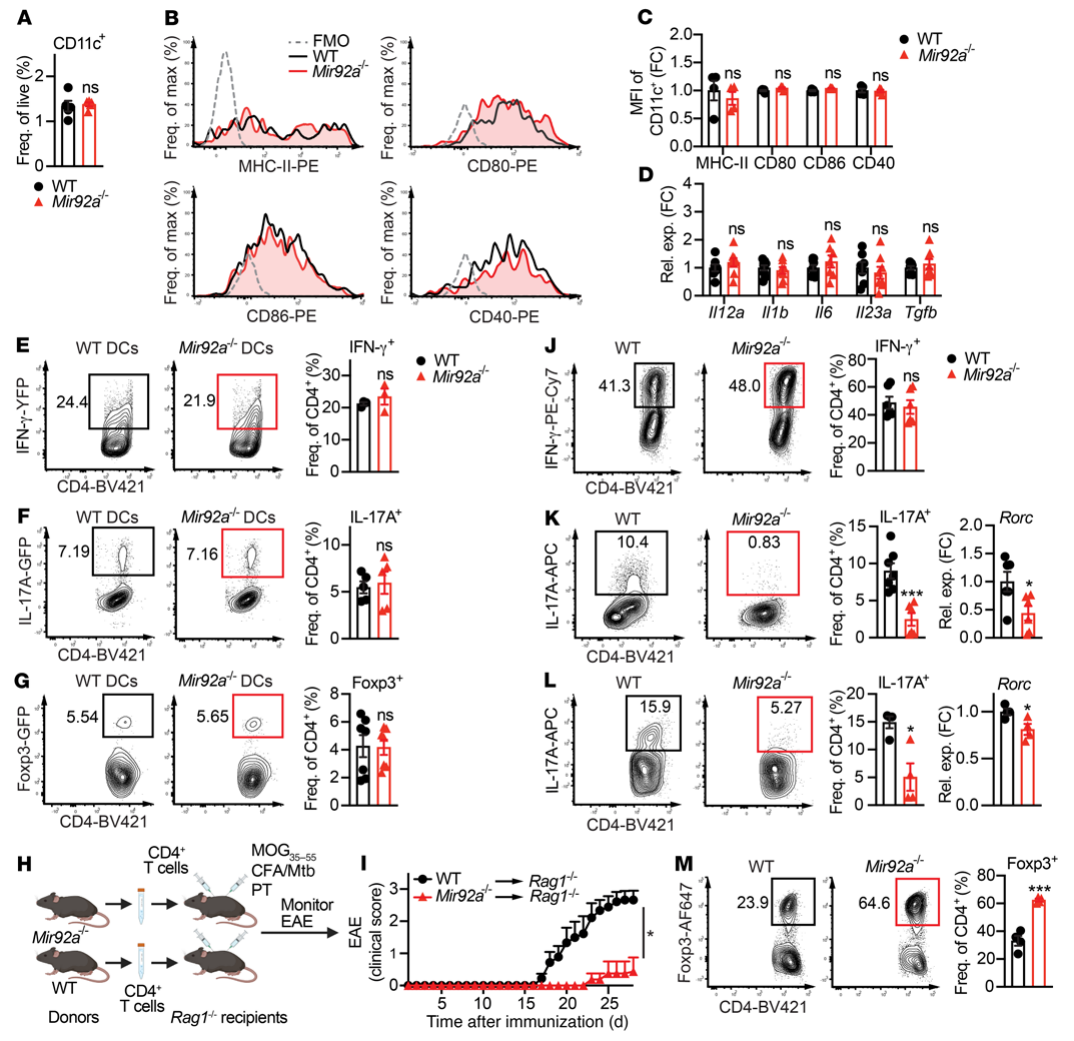
T cell–intrinsic miR-92a promotes EAE. (**A**) Frequency of CD11c^+^ splenic DCs from WT and *Mir92a^–/–^* mice at EAE onset (*n =* 5). (**B** and **C**) Representative flow cytometric histograms (**B**) and the MFI values (**C**) for MHC-II, CD80, CD86, and CD40 in DCs from these EAE mice (*n =* 4–5). MFIs are represented as the fold change relative to WT conditions. (**D**) qPCR analyses of Th-polarizing cytokines in DCs isolated from these EAE mice (*n =* 6–9). (**E**–**G**) Representative flow cytometric plots and frequencies of IFN-γ-YFP^+^ (*n =* 3) (**E**), IL-17A-GFP^+^ (*n =* 5) (**F**), and Foxp3-GFP^+^ (*n =* 7) (**G**) cells in WT naive CD4^+^ T cells cocultured with WT or *Mir92a^–/–^* DCs. (**H**) Adoptive transfer schematic. Total CD4^+^ T cells from WT and *Mir92a^–/–^* mice were transferred into *Rag1^–/–^* recipient mice, which were then immunized and monitored for EAE. Created with BioRender.com. (**I**) Clinical EAE scores of *Rag1^–/–^* recipient mice (*n =* 8–9) in (**H**). (**J**) Representative flow cytometric plots and frequencies of IFN-γ^+^ cells in Th1-polarized WT and *Mir92a^–/–^* naive CD4^+^ T cells (*n =* 6). (**K** and **L**) Representative flow cytometric plots and frequencies of IL-17A^+^ cells and qPCR analysis of *Rorc* expression in nonpathogenic Th17-polarized (*n =* 6–7) (**K**) or pathogenic Th17-polarized (*n =* 4) (**L**) WT and *Mir92a^–/–^* naive CD4^+^ T cells. (**M**) Representative flow cytometric plots and frequencies of Foxp3^+^ cells in Treg-polarized WT and *Mir92a^–/–^* naive CD4^+^ T cells (*n =* 4). Data are representative of 2–3 independent experiments and indicate the mean ± SEM. *P < 0.05 and ***P < 0.001, by unpaired, 2-tailed Student’s *t* test (**A**, **E**–**G**, and **J**–**M**), 1-way ANOVA with Šidák’s multiple-comparison test between WT and *Mir92a^–/–^* mice within each condition (**C** and **D**), or Mann-Whitney *U* test (**I**).

DCs are major regulators of T cell–mediated inflammation in EAE and MS (37). Therefore, we tested whether *Mir92a^–/–^* DCs could be driving the EAE attenuation and altered Th cell responses we observed in miR-92a*^–/–^* mice (Figure 1 and Supplemental Figure 2, A and B). We found no differences in CD11c^+^ DC frequencies between WT and miR-92a*^–/–^* mice during EAE (Figure 2A). Moreover, CD11c^+^ DCs from *Mir92a^–/–^* and WT mice expressed comparable levels of MHC-II and costimulatory molecules (CD80, CD86, and CD40) (Figure 2, B and C), as well as Th cell–polarizing cytokines (*Il12a*, *Il1b*, *Il6*, *Il23a*, and *Tgfb* (Figure 2D) during EAE. Furthermore, we found no differences in CD11c^+^ DCs from *Mir92a^–/–^* and WT mice for the expression of MHC-II and costimulatory molecules and Th cell–polarizing cytokines under LPS stimulation in vitro (Supplemental Figure 4, A–D). These data suggested that the ability of *Mir92a^–/–^* DCs to drive Th1, Th17, or Treg differentiation was unaltered. To directly test this possibility, we cocultured *Mir92a^–/–^* DCs or WT DCs with WT naive CD4^+^ T cells under Th1-, Th17-, or Treg-polarizing conditions. We observed no differences in the frequencies of these T cell subsets between cocultures with *Mir92a^–/–^* DCs or WT DCs (Figure 2, E–G). Together, these data suggested that DC-intrinsic miR-92a expression did not contribute to Th cell responses during EAE.

Therefore, we next assessed the CD4^+^ T cell–intrinsic role of miR-92a in EAE development. For this, we used an adoptive transfer model of EAE (27, 38), whereby total CD4^+^ T cells from *Mir92a^–/–^* mice or WT mice were transferred separately into recipient Rag1*^–/–^* mice followed by EAE induction (Figure 2H). We found that *Rag1^–/–^* recipients that received CD4^+^ T cells from *Mir92a^–/–^* mice developed attenuated EAE compared with *Rag1^–/–^* recipients that received WT cells (Figure 2I). Additionally, we used an effector CD4^+^ T cell–mediated adoptive transfer model of EAE, in which *Mir92a^–/–^* mice and WT mice were immunized with MOG_35–55_ and CFA/Mtb, followed by the isolation and transfer of effector CD4^+^ T cells from these immunized *Mir92a^–/–^* mice or WT mice into recipient naive WT C57BL/6 mice (Supplemental Figure 5A). Similar to the attenuated EAE seen in immunized *Rag1^–/–^* recipients that received CD4^+^ T cells from naive *Mir92a^–/–^* mice (Figure 2I), we found that WT recipients that received effector CD4^+^ T cells from immunized *Mir92a^–/–^* mice developed less severe EAE than did recipients that received cells from immunized WT mice (Supplemental Figure 5B). These results, altogether, recapitulated the attenuated EAE phenotype of *Mir92a^–/–^* mice (Figure 1, B–F) and suggested that miR-92a loss specifically within CD4^+^ T cells was sufficient to confer EAE attenuation.

We next directly investigated whether T cell–intrinsic miR-92a can influence Th cell development. We found the proliferative capacity and viability to be comparable between *Mir92a^–/–^* and WT naive CD4^+^ T cells following activation (Supplemental Figure 6, A and B). We then cultured *Mir92a^–/–^* naive CD4^+^ T cells under Th1-, Th17-, and Treg-polarizing conditions. Contrary to the reduced Th1 cell frequencies seen during EAE in *Mir92a^–/–^* mice (Figure 1H and Supplemental Figure 2, A and B), miR-92a loss did not affect IL-12–driven Th1 differentiation in vitro (Figure 2J). Interestingly, however, miR-92a loss impaired TGF-β– and IL-6–driven nonpathogenic Th17 differentiation (Figure 2K), as well as pathogenic Th17 differentiation induced by TGF-β, IL-6, IL-1β, and IL-23 in vitro (Figure 2L). These data were consistent with the reduction in Th17 cells observed in *Mir92a^–/–^* mice during EAE (Figure 1H and Supplemental Figure 2, A and B) and implicated miR-92a in promoting the development of both nonpathogenic and pathogenic Th17 cells. In contrast to the diminished Th17 cells, we found that miR-92a loss robustly enhanced Treg polarization in vitro (Figure 2M). This was consistent with the increased frequencies of Tregs found in *Mir92a^–/–^* mice during EAE (Figure 1I). Together, our findings thus far suggested that miR-92a exerted a CD4^+^ T cell–intrinsic function, whereby it promoted Th17 differentiation while limiting Treg differentiation to mediate EAE development.

### miR-92a inhibits Treg development by targeting Foxo1.

**Figure 3:**
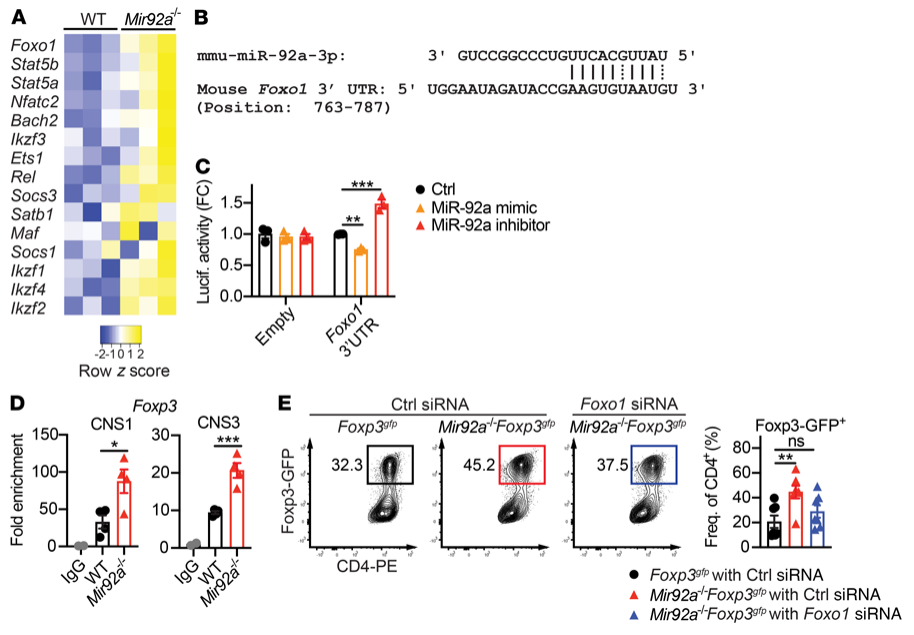
miR-92a inhibits Treg differentiation by targeting Foxo1. (**A**) Heatmap showing qPCR analyses of selected Treg/Th17-associated transcription factors in Treg-polarized WT and *Mir92a^–/–^* naive CD4^+^ T cells (*n =* 3). (**B**) In silico prediction analysis of complementary binding between miR-92a and the *Foxo1* 3′-UTR. (**C**) Luciferase activity in a HEK293T cell line cotransfected with luciferase plasmid containing no insert (empty) or a *Foxo1* 3′-UTR sequence, along with either LNA control (Ctrl), a miR-92a mimic, or a miR-92a inhibitor (*n =* 3). (**D**) ChIP analyses of Foxo1 binding to *Foxp3* CNS1 or CNS3 loci in Treg-polarized *Foxp3^gfp^* and *Mir92a^–/–^* *Foxp3^gfp^* naive CD4^+^ T cells (*n =* 4). Fold enrichment is shown relative to WT IgG conditions. (**E**) Representative flow cytometric plots and frequencies of Foxp3^+^ cells in *Foxp3^gfp^* and *Mir92a^–/–^* *Foxp3^gfp^* naive CD4^+^ T cells transfected with control or *Foxo1* siRNA and cultured under Treg-polarizing conditions (*n =* 7). Data are representative of 2–3 independent experiments and indicate the mean ± SEM. *P < 0.05, **P < 0.01, and ***P < 0.001, by 1-way ANOVA with Dunnet’s multiple-comparison test (**C** and **D**) or Šidák’s multiple-comparison test (**E**).

We next determined the underlying mechanisms by which miR-92a modulates Th cell differentiation. Within *Mir92a^–/–^* CD4^+^ T cells, we assessed the levels of Th lineage–associated transcription factors (TFs) known to regulate Treg and Th17 development (39–41). In agreement with enhanced Treg induction (Figure 2M), we found an overall upregulation of Treg-associated TFs (e.g., *Foxo1*, *Stat5a*, *Stat5b*) in *Mir92a^–/–^* CD4^+^ T cells compared with WT cells (Figure 3A). Among the Treg-associated TFs evaluated, Foxo1 (*Foxo1*) was robustly elevated (Figure 3A). Foxo1 is a critical transcription factor involved in Treg and Th17 cell development and function (42–46). Elevated *Foxo1* in *Mir92a^–/–^* T cells suggested it might be directly targeted by miR-92a. Because miRNAs primarily exert their function via complementary base pairing with the 3′-UTRs of target mRNAs, we searched for complementarity between the 3′-UTR of *Foxo1* and miR-92a using the RNA hybrid tool (47) and found a potential binding site (Figure 3B). To test whether *Foxo1* was a bona fide target of miR-92a, we performed a reporter assay by transfecting human embryonic kidney 293 T (HEK293T) cells with a *Foxo1* 3′-UTR luciferase construct in the presence of a miR-92a mimic or a miR-92a inhibitor. We found that the luciferase activity of the *Foxo1* 3′-UTR decreased in the presence of the miR-92a mimic, whereas luciferase activity was enhanced by the addition of the miR-92a inhibitor (Figure 3C). These results confirmed *Foxo1* as a bona fide target of miR-92a.

Since Foxo1 plays a critical role in early Treg lineage commitment (42, 44), we examined how elevated Foxo1 in *Mir92a^–/–^* CD4^+^ T cells might enable the increased Treg induction we observed (Figure 2M). Foxo1 has been shown to bind the conserved noncoding sequence regions 1 and 3 (CNS1 and CNS3) of the *Foxp3* locus to transactivate *Foxp3* within developing Tregs (44). In line with elevated *Foxo1* levels in *Mir92a^–/–^* Tregs, ChIP analyses revealed enhanced Foxo1 binding to these regions (Figure 3D). This increased Foxo1 activity, coupled with the increased Foxp3 expression observed in *Mir92a^–/–^* T cells, suggested that miR-92a may limit Treg induction by repressing *Foxo1*. Therefore, we assessed whether knockdown of *Foxo1* abrogated Treg promotion associated with miR-92a deficiency. We first confirmed that in vitro treatment of *Mir92a^–/–^* naive CD4^+^ T cells with *Foxo1* siRNA resulted in effective downregulation of Foxo1 protein (Supplemental Figure 7). Then, using *Mir92a^–/–^* *Foxp3^gfp^* reporter mice, we found that silencing *Foxo1* reduced Treg induction in *Mir92a^–/–^* T cells to a degree closer to that seen in WT cells (Figure 3E). Together, these results suggested that miR-92a inhibits Treg induction by targeting the Foxo1/Foxp3 axis.

### miR-92a promotes Treg acquisition of an inflammatory phenotype and impairs Treg suppressive function.

**Figure 4:**
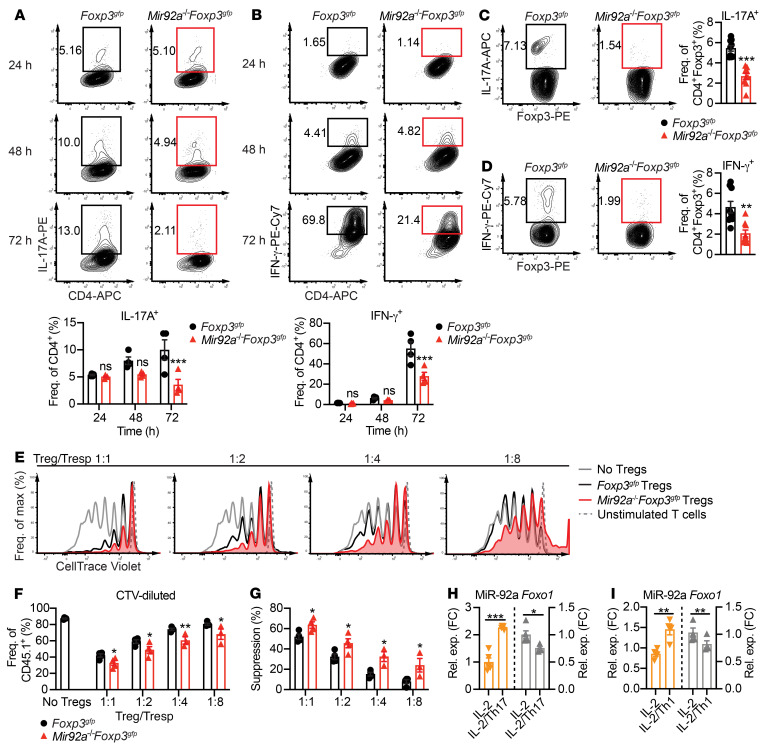
miR-92a promotes Treg acquisition of an inflammatory phenotype and impairs suppressive function. (**A** and **B**) Naive CD4^+^ T cells from *Foxp3^gfp^* and *Mir92a^–/–^* *Foxp3^gfp^* mice were differentiated in vitro into Tregs and sorted for GFP^+^ cells, followed by culturing with IL-2 and IL-1β/IL-6/IL-23 (**A**) or with IL-2 and IL-12/IL-6 (**B**) for 24, 48, and 72 hours. Representative flow cytometric plots and frequencies of IL-17A^+^ cells (**A**) and IFN-γ^+^ cells (**B**) are shown (*n =* 4). (**C** and **D**) Representative flow cytometric plots and frequencies of IL-17A^+^ Tregs (**C**) and IFN-γ^+^ Tregs (**D**) from the spleens of *Foxp3^gfp^* and *Mir92a^–/–^* *Foxp3^gfp^* mice at EAE onset (*n =* 9–10). (**E** and **F**) *Foxp3^gfp^* and *Mir92a^–/–^* *Foxp3^gfp^* mice were immunized with MOG_35–55_/CFA, and then GFP^+^ Tregs from dLNs and spleens were sorted at EAE onset and cocultured with CTV-labeled WT CD45.1^+^ naive CD4^+^ Tresp cells and APCs. Representative flow cytometric plots (**E**) and frequencies (**F**) of CTV-labeled WT Tresp cells at the indicated Treg/Tresp ratios (*n =* 3–4). (**G**) Percentage of suppression calculated for the *Foxp3^gfp^* and *Mir92a^–/–^* *Foxp3^gfp^* Tregs in **F**. (**H** and **I**) Naive CD4^+^ T cells from *Foxp3^gfp^* mice were differentiated into Tregs and then sorted for GFP^+^ cells, followed by culturing with IL-2 alone or IL-2 plus either IL-1β/IL-6/IL-23 (IL-2/Th17) (**H**) or IL-6/IL-12 (IL-2/Th1) (**I**) for 24 hours. qPCR analyses of miR-92a (left) and *Foxo1* (right) are shown (*n =* 4–5). Data are representative of 2–3 independent experiments and indicate the mean ± SEM. *P < 0.05, **P < 0.01, and ***P < 0.001, by unpaired, 2-tailed Student’s *t* test (**C**, **D**, **H**, and **I**) or 1-way ANOVA with Šidák’s multiple-comparison test between WT and *Mir92a^–/–^* mice within each condition (**A**, **B**, **F**, and **G**).

In addition to promoting Treg development, Foxo1 also maintains Treg homeostasis and function by preventing Tregs from acquiring an inflammatory phenotype (42, 45). When exposed to an inflammatory cytokine milieu, Tregs become dysfunctional and convert into Th17- or Th1-like cells expressing effector cytokines including IL-17A (48, 49) and IFN-γ (50). Such defects in suppressive Tregs have been associated with multiple inflammatory and autoimmune diseases, including EAE (6) and MS (9, 10). Because miR-92a loss led to higher Foxo1 expression, we asked whether miR-92a loss would alter Treg acquisition of an inflammatory phenotype. To test this, naive CD4^+^ T cells from WT *Foxp3^gfp^* or *Mir92a^–/–^* *Foxp3^gfp^* mice were differentiated into Tregs in vitro. FACS-sorted Foxp3-GFP^+^ Tregs were subsequently cultured in vitro with IL-2 alongside Th1- or Th17-polarizing cytokines known to destabilize Tregs (51). As predicted, we found that exposure of WT Tregs to Th17- or Th1-inducing cytokines led to increased IL-17A or IFN-γ, respectively (Figure 4, A and B). However, *Mir92a^–/–^* Tregs remained relatively resistant to these cytokine-mediated changes (Figure 4, A and B). Of note, *Mir92a^–/–^* Foxp3-GFP^+^ and WT Foxp3-GFP^+^ Tregs cultured with IL-2 alone did not differ in their ability to upregulate IL-17A and IFN-γ at any of the time points examined (Supplemental Figure 8, A and B). We next sought to determine whether this resistance of *Mir92a^–/–^* Tregs to acquiring an inflammatory phenotype also occurred in vivo during EAE. Consistent with our observations in vitro, we found that Tregs from *Mir92a^–/–^* *Foxp3^gfp^* mice expressed less IL-17A and IFN-γ than did those from WT mice during EAE (Figure 4, C and D).

Since acquisition of an inflammatory phenotype in Tregs is associated with an impaired suppressive function (52), we then tested whether Tregs from *Mir92a^–/–^* mice during EAE exhibit an enhanced suppressive function. For this, we induced EAE in *Foxp3^gfp^* and *Mir92a^–/–^* *Foxp3^gfp^* mice, FACS-sorted Foxp3-GFP^+^ Tregs from these mice, and then cocultured them with different ratios of CellTrace Violet–labeled (CTV-labeled) naive WT CD4^+^ T responder (Tresp) cells. We found that Tresp cells cultured with Tregs from *Mir92a^–/–^* *Foxp3^gfp^* mice proliferated significantly less than did those cultured with Tregs from *Foxp3^gfp^* mice at all ratios tested (1:1 to 1:8) (Figure 4, E and F). As such, Tregs from *Mir92a^–/–^* *Foxp3^gfp^* mice demonstrated an enhanced suppressive ability (Figure 4G). Next, we asked whether exposure of Tregs to an inflammatory cytokine milieu could lead to increased miR-92a, and how this increased miR-92a might be linked to acquisition of an inflammatory Treg phenotype. For this, we cultured naive CD4^+^ T cells from WT *Foxp3^gfp^* mice under Treg-polarizing conditions, FACS-sorted Foxp3-GFP^+^ Tregs, and then exposed these Tregs in vitro to Th17- and Th1-inducing cytokines. We found that these inflammatory cytokines resulted in robust upregulation of miR-92a in WT Tregs (Figure 4, H and I). Correspondingly, miR-92a upregulation was associated with downregulation of the miR-92a target *Foxo1* in WT Tregs (Figure 4, H and I). These data suggested that miR-92a targeting of *Foxo1* also promoted the acquisition of an inflammatory phenotype in Tregs. Together, our results demonstrated that miR-92a may negatively regulate not only the development of Tregs, but also their suppressive function during CNS autoimmunity.

Although we found that T cell–intrinsic miR-92a loss did not alter Th1 cell differentiation in vitro (Figure 2J), we observed that miR-92a loss was associated with significantly decreased IFN-γ^+^ Th1 responses in vivo during EAE (Figure 1H). Given our data demonstrating that miR-92a loss promoted Treg induction (Figure 2M) and Treg suppressive activity (Figure 4, E–G), and given the well-established role for Tregs in limiting Th1 induction (52), we asked whether enhanced Treg responses in *Mir92a^–/–^* mice were responsible for their decreased Th1 responses in vivo during EAE (Figure 1H). To address this, we treated *Mir92a^–/–^* mice with anti-CD25 monoclonal antibodies to deplete Tregs during EAE (53). Anti-CD25 treatment effectively reduced Foxp3^+^ Treg frequencies compared with those seen in isotype-treated *Mir92a^–/–^* mice (Supplemental Figure 9A). Importantly, anti-CD25 treatment also led to enhanced IFN-γ^+^ Th1 responses in *Mir92a^–/–^* mice to levels comparable to those observed in WT mice (Supplemental Figure 9B). These results suggested that, although miR-92a loss itself may not directly alter the differentiation of Th1 cells, it may still limit Th1 responses during EAE indirectly by affecting Treg responses.

### miR-92a promotes Th17 development by targeting Foxo1.

**Figure 5:**
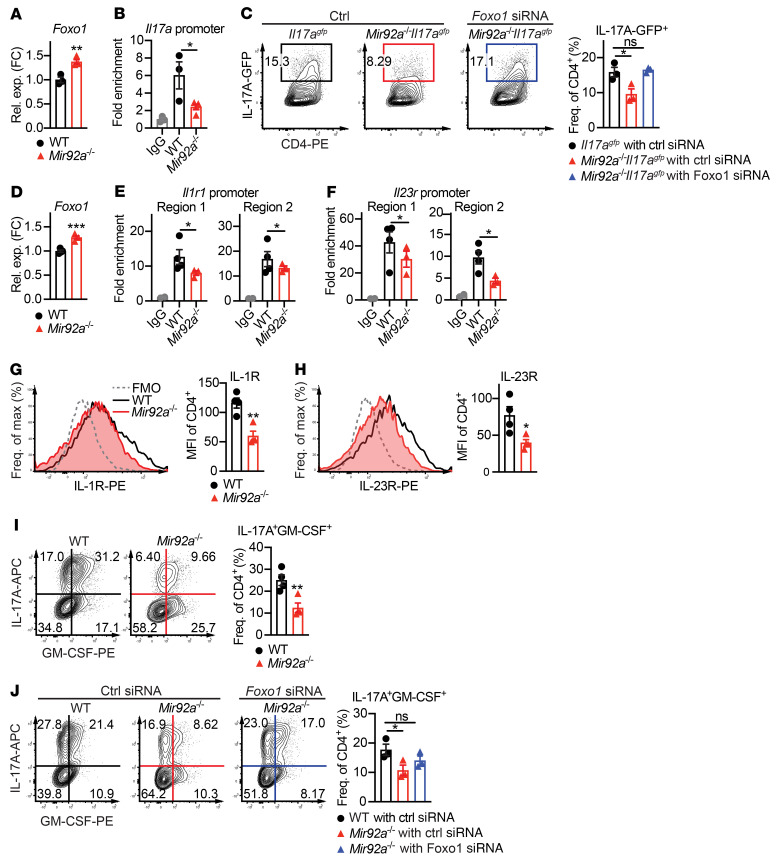
miR-92a promotes nonpathogenic and pathogenic Th17 development by targeting Foxo1. (**A**) qPCR analysis of *Foxo1* expression in nonpathogenic Th17-polarized WT and *Mir92a^–/–^* naive CD4^+^ T cells (*n =* 3–4). (**B**) ChIP analysis of RORγt binding to the *Il17a* locus in nonpathogenic Th17-polarized WT and *Mir92a^–/–^* naive CD4^+^ T cells (*n =* 3–4). (**C**) Representative flow cytometric plots and frequencies of IL-17A^–^GFP^+^ cells in *Il17a^gfp^* and *Mir92a^–/–^* *Il17a^gfp^* naive CD4^+^ T cells, transfected in vitro with control or *Foxo1* siRNA and then cultured under nonpathogenic Th17-polarizing conditions (*n =* 3). (**D**) qPCR analysis of *Foxo1* expression in pathogenic Th17-polarized WT and *Mir92a^–/–^* naive CD4^+^ T cells (*n =* 4). (**E** and **F**) ChIP analyses of RORγt binding to the *Il1r1* promoter loci (**E**) and *Il23r* promoter loci (**F**) in pathogenic Th17-polarized WT and *Mir92a^–/–^* naive CD4^+^ T cells (*n =* 3–4). (**G** and **H**) Representative flow cytometric histograms and MFIs of IL-1R (**G**) and IL-23R (**H**) in pathogenic Th17-polarized WT and *Mir92a^–/–^* CD4^+^ T cells (*n =* 4). MFI values shown were obtained after subtracting the MFI values of fluorescence minus one (FMO) controls for IL-1R or IL-23R. (**I**) Representative flow cytometric plots and frequencies of IL-17A^+^GM-CSF^+^ cells from **G** and **H** (*n =* 4). (**J**) Representative flow cytometric plots and frequencies of IL-17A^+^GM-CSF^+^ cells in WT and *Mir92a^–/–^* naive CD4^+^ T cells transfected with control or *Foxo1* siRNA followed by culturing under pathogenic Th17-polarizing conditions (*n =* 3). Fold enrichment is shown relative to WT IgG conditions (**B**, **G**, and **H**). Data are representative of 2 independent experiments and indicate the mean ± SEM. *P < 0.05, **P < 0.01, and ***P < 0.001, by unpaired, 2-tailed Student’s *t* test (**A**, **D**, **G**–**I**) or 1-way ANOVA with Dunnet’s multiple-comparison test (**B**, **C**, **E**, **F**, and **J**).

We next examined whether miR-92a targeting of *Foxo1* might promote nonpathogenic and pathogenic Th17 cell differentiation. We first confirmed that nonpathogenic Th17-polarized *Mir92a^–/–^* CD4^+^ T cells also expressed increased *Foxo1* levels (Figure 5A). Within developing Th17 cells, RORγt induces the expression of the Th17 signature cytokine IL-17A (16). While PI3K/AKT signaling is required for RORγt nuclear translocation and subsequent IL-17A induction (54), Foxo1 has been shown to inhibit the RORγt-mediated transactivation of IL-17A by preventing RORγt from binding to the *Il17a* locus (43). Thus, we examined whether *Mir92a^–/–^* Th17 cells with elevated Foxo1 showed altered RORγt binding to the *Il17a* locus. Accordingly, ChIP analyses showed such decreased RORγt binding (Figure 5B), consistent with enhanced Foxo1 activity in these cells. This increased Foxo1 activity, coupled with the decreased Th17 differentiation we observed earlier in *Mir92a^–/–^* T cells (Figure 2J), suggested that miR-92a may promote nonpathogenic Th17 cells by repressing *Foxo1*. Indeed, using *Mir92a^–/–^* *Il17a^gfp^* reporter mice, we found that silencing *Foxo1* restored IL-17A levels in *Mir92a^–/–^* CD4^+^ T cells closer to its levels in WT cells (Figure 5C).

Analogous to our findings in nonpathogenic Th17 conditions, we also detected elevated *Foxo1* in pathogenic Th17-polarized *Mir92a^–/–^* CD4^+^ T cells in vitro (Figure 5D). In such pathogenic Th17 cells differentiated in the presence of IL-1β and IL-23, RORγt can induce the expression of IL-1R and IL-23R, both of which drive GM-CSF induction during EAE (21, 22). Furthermore, Foxo1 has been shown to inhibit this RORγt-mediated transactivation of *Il1r1* and *Il23r* (26, 43, 46) by preventing RORγt from binding to the *Il1r1* and *Il23r* promoters (21, 22). In line with elevated *Foxo1*, our ChIP analyses revealed diminished RORγt binding to these promoters in pathogenic Th17-polarized *Mir92a^–/–^* cells (Figure 5, E and F). Accordingly, we found that pathogenic Th17-polarized *Mir92a^–/–^* CD4^+^ T cells expressed less IL-1R (Figure 5G) and IL-23R (Figure 5H), alongside correspondingly diminished GM-CSF (Figure 5I). These results were consistent with our observations during EAE (Figure 1H and Supplemental Figure 2, A and B). Furthermore, we found that silencing *Foxo1* in *Mir92a^–/–^* CD4^+^ T cells restored the levels of IL-17A and GM-CSF closer to those detected in WT cells (Figure 5J). Together, these data suggest that miR-92a promotes pathogenic Th17 differentiation and GM-CSF induction by targeting *Foxo1*, relieving the suppressive effect of Foxo1 on IL-1R and IL-23R.

### miR-92a inhibitor treatment ameliorates EAE.

**Figure 6:**
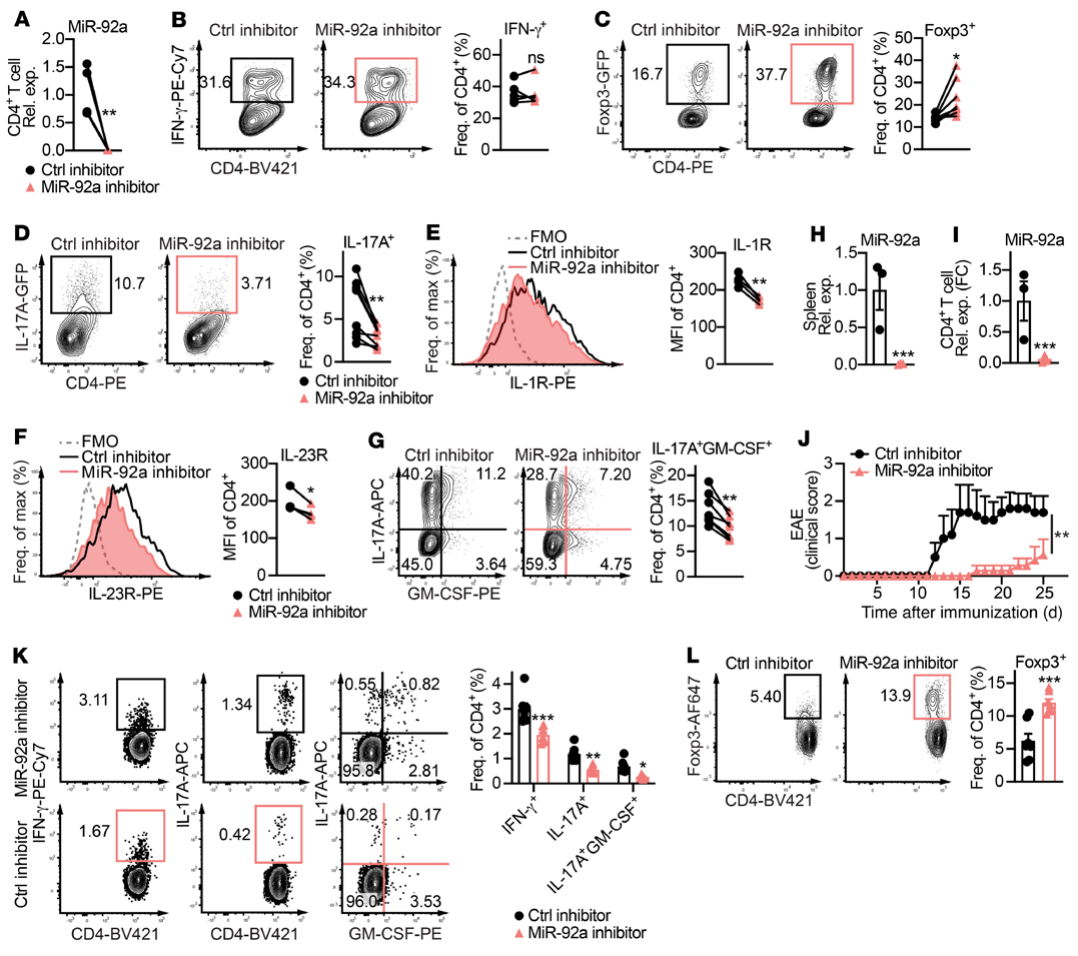
miR-92a inhibitor treatment ameliorates EAE. (**A**) qPCR analysis of miR-92a in splenic CD4^+^ T cells cultured in vitro with miR-92a or a control inhibitor (*n =* 5). (**B**–**D**) Representative flow cytometric plots and frequencies of IFN-γ^+^ (*n =* 5) (**B**), Foxp3^+^ (*n =* 7) (**C**), and IL-17A^+^ (*n =* 8) (**D**) cells among WT naive CD4^+^ T cells cultured under either Th1- (**B**), Treg- (**C**), or (**D**) nonpathogenic Th17-polarizing conditions with miR-92a or a control inhibitor, respectively. (**E** and **F**) Representative flow cytometric histograms and MFIs of IL-1R (**E**) and IL-23R (**F**) in pathogenic Th17-polarized WT naive CD4^+^ T cells cultured with a control or miR-92a inhibitor (*n =* 4). MFI values shown were obtained after subtracting the MFI values of the FMO controls for IL-1R or IL-23R. (**G**) Representative flow cytometric plots and frequencies of IL-17A^+^GM-CSF^+^ cells under the same conditions as in **E** and **F**. (**H** and **I**) qPCR analyses of miR-92a levels in total splenocytes (**H**) and splenic CD4^+^ T cells (**I**) from control and miR-92a inhibitor–treated mice (*n =* 3). (**J**) Clinical EAE scores for treated WT mice (*n =* 5–7). (**K** and **L**) Representative flow cytometric plots and frequencies of IFN-γ^+^, IL-17A^+^, GM-CSF^+^ (**K**), and Foxp3^+^ (**L**), splenic CD4^+^ T cells from treated mice at EAE onset (*n =* 6). Data are representative of 2 or more independent experiments and indicate the mean ± SEM. *P < 0.05, **P < 0.01, and ***P < 0.001, by paired, 2-tailed Student’s *t* test (**A**–**G**), unpaired, 2-tailed Student’s *t* test (**H**, **I**, and **L**), Mann-Whitney *U* test (**J**), or 1-way ANOVA with Šidák’s multiple-comparison test between control inhibitor and miR-92a inhibitor treatment within each condition (**K**).

Because our data thus far suggested that miR-92a promotes EAE by inhibiting Tregs and by promoting Th17 cells, we sought to explore the therapeutic efficacy of miR-92a inhibition during EAE. We and others have previously leveraged miRNA-based therapeutics in inflammation and CNS autoimmunity (27, 28, 55, 56). We validated a locked nucleic acid–modified (LNA-modified) miR-92a Power Inhibitor in vitro and found that it ablated miR-92a expression in WT CD4^+^ T cells (Figure 6A). Next, we tested the effect of the miR-92a inhibitor on Th1, Treg, and Th17 differentiation in WT naive CD4^+^ T cells in vitro. Consistent with unaltered Th1 differentiation in *Mir92a^–/–^* CD4^+^ T cells (Figure 2J), the miR-92a inhibitor had no apparent effect (Figure 6B). However, analogous to elevated Treg differentiation in *Mir92a^–/–^* CD4^+^ T cells (Figure 2M), we found that miR-92a silencing enhanced Treg frequencies (Figure 6C). Conversely, miR-92a silencing reduced nonpathogenic Th17 cell differentiation (Figure 6D). We also found that miR-92a silencing led to impaired pathogenic Th17 differentiation, marked by a concomitant decrease in IL-1R, IL-23R, and GM-CSF (Figure 6, E–G). These Th17 data were again in line with impaired Th17 differentiation in *Mir92a^–/–^* CD4^+^ T cells (Figure 2, K and L). Finally, we tested the ability of the miR-92a inhibitor to alter EAE severity. For this, we induced EAE in WT mice and treated them with a miR-92a inhibitor every alternate day, 5 times beginning on day 3 after immunization. The treatment effectively ablated miR-92a in splenocytes and CD4^+^ T cells in vivo (Figure 6, H and I). Most important, miR-92a inhibition delayed the onset of EAE and reduced disease progression (Figure 6J). In agreement with our data in *Mir92a^–/–^* mice (Figure 1 and Supplemental Figure 2), this EAE attenuation was associated with significantly reduced frequencies of IFN-γ^+^, IL-17A^+^, and IL-17A^+^GM-CSF^+^ CD4^+^ T cells (Figure 6K) and an increased fraction of Foxp3^+^ Tregs (Figure 6L). Together, these results demonstrated that miR-92a inhibitor treatment ameliorated EAE by altering the balance of inflammatory and regulatory T cells.

### miR-92a inhibition promotes Treg induction and inhibits Th17 differentiation in CD4^+^ T cells from patients with MS.

**Figure 7:**
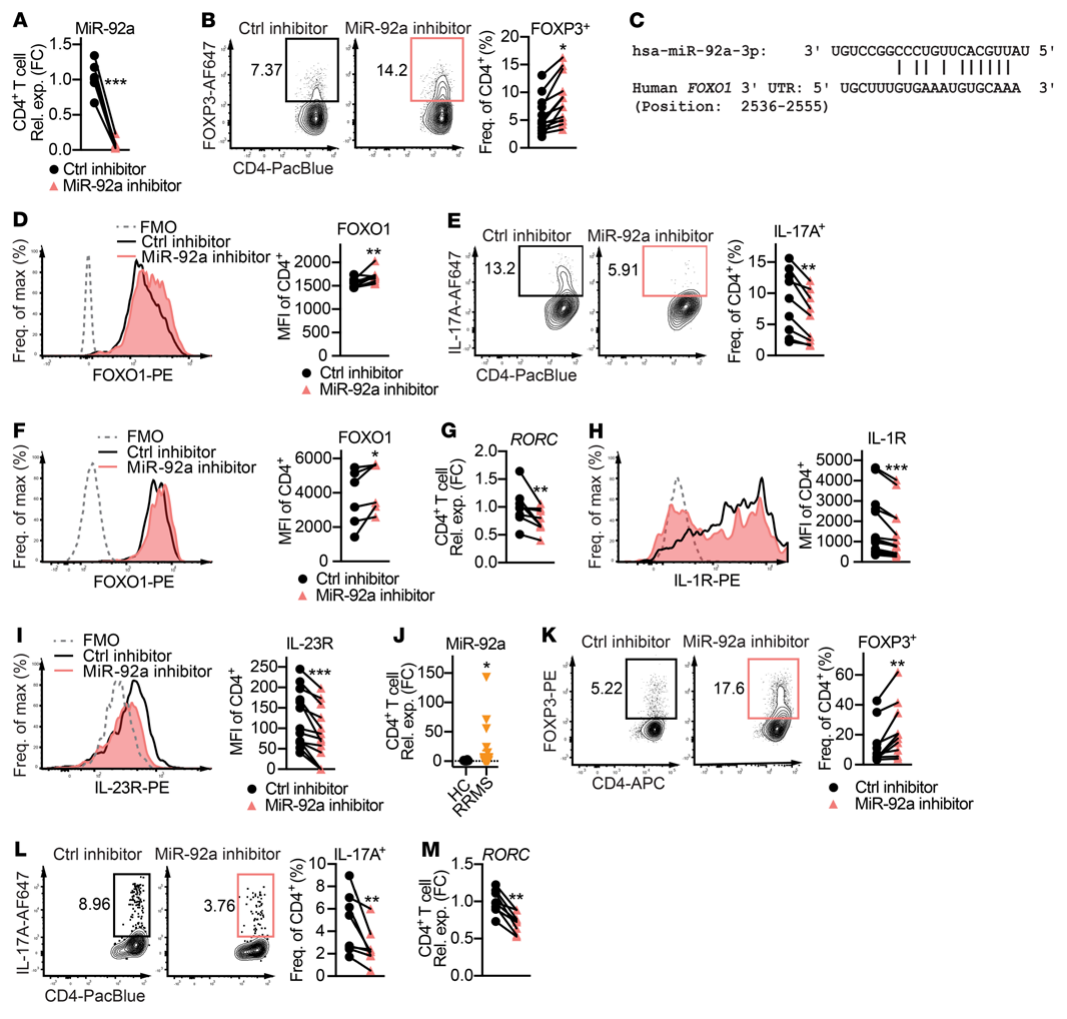
miR-92a inhibitor promotes Treg induction and inhibits Th17 differentiation in CD4^+^ T cells from patients with MS. (**A**) qPCR analysis of miR-92a expression in HC naive CD4^+^ T cells cultured with a control or miR-92a inhibitor (*n =* 7). (**B**) Representative flow cytometric plots and frequencies of Foxp3^+^ cells in Treg-polarized HC naive CD4^+^ T cells cultured with a control or miR-92a inhibitor (*n =* 13). (**C**) In silico prediction of the complementary miR-92a and *FOXO1* 3′-UTR sequences. (**D**) Flow cytometric histograms and MFIs of Foxo1 in Treg-polarized HC naive CD4^+^ T cells cultured with a control or miR-92a inhibitor (*n =* 10). (**E**) Representative flow cytometric plots and frequencies of IL-17A in Th17-polarized HC naive CD4^+^ T cells cultured with either inhibitor (*n =* 9). (**F**) Representative flow cytometric histograms and MFIs of Foxo1 in these cells (*n =* 7). (**G**) qPCR analysis of *RORC* expression in these cells (*n =* 8). (**H** and **I**) Representative flow cytometric histograms and MFIs of IL-1R (**H**) and IL-23R (**I**) in these cells (*n =* 14). MFI values shown were obtained after subtracting the MFI values of the FMO controls for IL-1R or IL-23R. (**J**) qPCR analysis of miR-92a levels in total CD4^+^ T cells from HCs (*n =* 23) and untreated patients with RRMS (*n =* 28). (**K**) Representative flow cytometric plots and frequencies of Foxp3 in Treg-polarized MS naive CD4^+^ T cells cultured with a control or miR-92a inhibitor (*n =* 10). (**L**) Representative flow cytometric plots and frequencies of IL-17A^+^ cells in Th17-polarized MS naive CD4^+^ T cells cultured with either inhibitor (*n =* 8). (**M**) qPCR analysis of *RORC* expression in these cells. Data are representative of 2 or more independent experiments and indicate the mean ± SEM. *P < 0.05, **P < 0.01, and ***P < 0.001, by Wilcoxon signed-rank test (**A**, **B**, **D**–**I**, and **K**–**M**) or Mann-Whitney *U* test (**J**). PacBlue, Pacific blue stain.

Next, we asked whether the disease-promoting miR-92a mechanisms we observed in mice also occurred in human T cells. We first demonstrated that the miR-92a inhibitor robustly abolished miR-92a expression in total CD4^+^ T cells (Figure 7A) from HC donors (Supplemental Table 1). Consistent with our data in mice (Figure 6B), we found that miR-92a silencing had no effect on Th1 differentiation in HC CD4^+^ T cells (Supplemental Figure 10A). Excitingly, however, we found that miR-92a silencing promoted Treg differentiation in HC CD4^+^ T cells (Figure 7B). Given that miR-92a targets *Foxo1* to modulate Treg differentiation in mice, we asked whether miR-92a acted similarly in human Tregs. Analogous to mice, we identified a potential complementary binding site between miR-92a and the human *FOXO1* 3′-UTR using the RNA hybrid tool (ref. 47 and Figure 7C). In support of direct binding, we observed that enhanced Treg differentiation by miR-92a inhibitor was accompanied by increased Foxo1 (Figure 7D). In line with our mouse findings, we also observed that miR-92a silencing significantly restricted Th17 differentiation in HC CD4^+^ T cells (Figure 7E), and this was accompanied by elevated Foxo1 and reduced *RORC* expression (Figure 7, F and G). These results indicated that miR-92a targeting of *FOXO1* also promoted human Th17 differentiation. As in mice, RORC-induced expression of IL-1R and IL-23R is essential for human Th17 development (57, 58). Indeed, miR-92a inhibitor treatment also reduced IL-1R and IL-23R in Th17-polarized HC CD4^+^ T cells (Figure 7, H and I). Altogether, these data suggested miR-92a inhibits Treg development while promoting Th17 differentiation in human CD4^+^ T cells.

Because our data supported a CD4^+^ T cell–intrinsic, pathogenic role for miR-92a, we asked whether miR-92a levels were altered specifically within the CD4^+^ T cells from patients with MS. For this, we isolated total CD4^+^ T cells from the PBMCs of untreated patients with relapsing-remitting MS (RRMS) and of sex- and age-matched HC donors (Supplemental Table 2), and measured miR-92a by quantitative PCR (qPCR). We found that miR-92a was significantly elevated in total CD4^+^ T cells from untreated MS patients compared with HC counterparts (Figure 7J). Finally, we examined whether miR-92a silencing could alter Treg and Th17 differentiation in CD4^+^ T cells from patients with MS (Supplemental Table 3). Consistent with our findings in HCs (Supplemental Figure 10A and Figure 7, B and E), we found that silencing of miR-92a in MS patients’ CD4^+^ T cells did not alter Th1 differentiation (Supplemental Figure 10B), yet promoted Treg induction (Figure 7K) and limited Th17 differentiation (Figure 7L). In parallel to our HC data, miR-92a inhibitor treatment in Th17-polarized MS patients’ CD4^+^ T cells also resulted in reduced expression of *RORC* (Figure 7M), IL-1R, and IL-23R (Supplemental Figure 10, C and D). Together, these results showed that miR-92a was elevated in CD4^+^ T cells from patients with MS and that miR-92a silencing in T cells of patients with MS effectively shifted the Th cell balance toward Tregs and away from Th17 cells.

## Discussion

Although numerous studies have described the disequilibrium between Tregs and Th17 cells in EAE and MS (3), its underlying mechanisms remain largely unknown. The miRNA regulation of T cell development and function in autoimmunity has recently become an area of intensive study. Previous work by us and others has identified miRNA pathways that either regulate Tregs (59–61) or inflammatory T cells (27–29, 38, 62), but typically not both, in EAE. Toward developing an effective miRNA-based therapeutic approach for CNS autoimmunity, it is imperative to identify specific miRNA pathways that are not only dysregulated in MS, but also capable of modulating both Tregs and Th17 cells in humans. Here, we report that miR-92a is a powerful, clinically relevant target that simultaneously modulates both Treg and Th17 cell responses (and indirectly regulates Th1 responses) to promote EAE and MS pathogenesis. Our findings also suggest miR-92a silencing as a unique 2-pronged approach to address the Treg/Th17 imbalance in CNS autoimmunity.

Previous work by us and others has identified miR-92a as one of the most significantly increased miRNAs in patients, with MS with direct links to clinical parameters (33, 35). miR-92a has also been found to be elevated in the cerebral white matter of patients with MS (32). In addition, another study found a trend toward a positive correlation between exosomal miR-92a and lymphocyte counts in patients with MS during relapse (63). Altogether, these studies had suggested a potential disease-promoting role for miR-92a in MS. This microRNA belongs to the miR-17~92 cluster (miR-17, miR-18a, miR-19a, miR-20a, miR-19b-1, and miR-92a). The miR-17~92 cluster has mostly been studied in cancer (64), and has only recently been implicated in autoimmunity (61, 65–67). Of note, T cell–specific deletion of the entire miR-17~92 cluster has been shown to alter T cell function in EAE (61, 65, 67). Although these studies have highlighted the whole miR-17~92 cluster in modulating inflammation, the functional relevance of altered miR-92a in MS and the specific role of miR-92a in Th cell responses during CNS autoimmunity have remained unknown. This gap has been critical to fill, given miR-92a’s robust clinical associations with MS pathology. Consistent with elevated miR-92a that has been seen in sera from patients with MS (33), we found that miR-92a was elevated in mice with EAE and that its loss conferred striking protection against EAE. Mechanistically, we characterize how T cell–intrinsic miR-92a drives EAE by limiting the induction of Tregs and their suppressive activity, while promoting Th17 cell differentiation. Our results also identify *Foxo1* as a key target of miR-92a in these processes.

Foxo1 is indispensable for Treg development and for preventing Treg dysfunction in inflammatory microenvironments (10, 42, 44, 45). In MS specifically, previous studies have shown that defective suppressive function of patients’ Tregs is associated with impaired Foxo1 signaling and increased IFN-γ (9, 10), and reversal of this impaired Foxo1 signaling restores Treg-suppressive activity (10). Our report suggests that by repressing Foxo1, miR-92a not only prevents de novo Treg development from naive CD4^+^ T cells, but also negatively impacts the suppressive function of preexisting Tregs within inflammatory contexts. Thus, our present study implicates a potential role for miR-92a in defective Foxo1 signaling and Treg dysfunction that has been observed in patients with MS.

Because our data show that miR-92a loss or miR-92a inhibition does not appear to directly regulate Th1 cell differentiation in vitro, the reduced Th1 cell frequency associated with EAE attenuation in *Mir92a^–/–^* mice could have been due to enhanced functional Treg responses. In support of this idea, our results demonstrated that depletion of Tregs in *Mir-92a^–/–^* mice rescued impaired IFN-γ^+^ Th1 cell responses in *Mir-92a^–/–^* mice during EAE. Interestingly, one report found that artificial overexpression of miR-92a seems to affect IFN-γ expression in splenocytes (68). Given that miRNA overexpression can result in nonphysiological targeting of mRNAs (69) and that the authors did not perform direct target validation or miR-92a functional studies during the EAE disease course, it is challenging to interpret these results in the context of our study. Our study more closely reveals native miR-92a function using *Mir92a^–/–^* mice and suggests that physiological levels of miR-92a did not appear to directly regulate Th1 cells. Similarly, we found the therapeutic benefit of miR-92a silencing appeared to be mediated through direct effects on Tregs and Th17 cells, with indirect effects on Th1 cells via Tregs.

To further perpetuate CNS autoimmunity, our data show that miR-92a supported inflammatory Th17 cell responses also by inhibiting *Foxo1*. In contrast to its positive role in Treg biology, Foxo1 negatively regulates Th17 cells at multiple levels (26, 43, 46). Specifically, Foxo1 limits nonpathogenic Th17 differentiation by inhibiting RORγt induction of IL-17A (43) and restricts pathogenic Th17 differentiation by preventing IL-1R/IL-23R signaling and induction of GM-CSF (26, 46). Our results demonstrate that miR-92a repression of *Foxo1* promoted both of these nonpathogenic and pathogenic Th17 processes. Together, our findings highlight a functional role for miR-92a in the reciprocal regulation of Tregs and inflammatory Th17 responses that drive CNS autoimmunity.

Although several antiinflammatory drugs are approved for the treatment of MS, they are insufficient to maintain long-term immune tolerance and halt progression in most patients (70). Antibodies targeting the inflammatory arm of the immune system alone have yielded disappointing results. Specifically, secukinumab, which targets IL-17A (71, 72), and ustekinumab, which targets the IL-12p40 subunit shared by both IL-12 and IL-23 (73), have not shown sufficient efficacy in clinical trials. There remains an unmet clinical need for new therapeutic strategies that restore the Treg/Th17 equilibrium by simultaneously promoting Treg responses and limiting pathogenic T cell responses in MS. We demonstrate that miR-92a silencing therapy by an LNA-modified miR-92a inhibitor robustly attenuated EAE and was associated with increased Tregs and diminished Th17 and Th1 cells. Importantly, we show that miR-92a was elevated in CD4^+^ T cells from patients with MS and that miR-92a silencing also effectively promoted both Treg induction and impaired Th17 differentiation in cultured T cells from both HCs and patients with MS. We therefore speculate that miR-92a inhibition may offer advantages compared with anti–IL-17 monoclonal antibody–based therapies in MS.

Interestingly, elevated plasma miR-92a has been found in patients with systemic lupus erythematosus (SLE) (74), as well as in those with scleroderma (75) or ulcerative colitis (UC) (76). Although SLE, scleroderma, and UC differ from MS in their pathophysiology and types of organs involved, Th17 cells are found to be increased in these patients and appear to be associated with disease activity (77–79). In addition, Tregs also play a critical role in suppressing the inflammatory responses in all these diseases (80–82). Furthermore, the imbalance between Treg and Th17 cell subsets has been reported in patients with these diseases (83–85). Given the clinical data associating Treg/Th17 imbalance with SLE, scleroderma, and UC and the robust role of miR-92a in modulating the Treg/Th17 balance, it may be of interest to explore miR-92a in these contexts. We speculate that future studies evaluating the functional role of miR-92a and miR-92a silencing in preclinical models of these disorders might be productive lines of investigation.

With the advent of the first approved mRNA vaccines and recent FDA-approved siRNA therapeutics such as patisiran and inotersen (86), alongside ongoing preclinical and clinical miRNA-based therapies (87), RNA targets are emerging as next-generation medicine. Regarding the safety and translational potential of miR-92a inhibition for MS in particular, our data suggest that the miR-92a inhibitor did not show any apparent toxicity in the treated C57BL/6 mice, and miR-92a-deficient mice are viable, fertile, and appear to develop normally (36). In fact, an LNA-based miR-92a inhibitor, MRG-110, was recently shown to be safe and well tolerated by healthy volunteers in a phase I clinical trial for cardiovascular indications (ClinicalTrials.gov NCT03603431; ref. 88). Moreover, infusion of MRG-110 can effectively silence miR-92a expression in the peripheral blood compartment of healthy individuals (European Clinical Trials no. 2017-004180-12; ref. 89). These clinical developments in RNA medicine support miR-92a inhibition as an attractive, actionable approach that could be readily tested in patients with MS. However, several concerns must be addressed. Previous studies have implicated a role for miR-92a in other pathobiological processes including type 1 diabetes (T1D) (90, 91), neurogenesis (92), cancer (25, 64, 93), as well as TLR (94) and estrogen receptor signaling (95). These studies were performed using either the entire miR-17~92 cluster–deficient mice (91, 92) or without the use of *Mir92a^–/–^* mice or miR-92a inhibitors in vivo (90, 94, 95). Therefore, further investigations using *Mir92a^–/–^* mice and miR-92a inhibitor treatment of preclinical animal models of disease and patients’ samples may help us to better understand the specific role of miR-92a in these processes. Given the pleiotropic roles of the miR-17~92 cluster and miR-92a in various physiological and disease contexts (25, 64, 90–95) and that early trials with an miR-92a inhibitor only monitored healthy volunteers for a limited length of time after the treatment (88, 89), it would be important to conduct a broad assessment of the long-term impact of miR-92a inhibition for potential adverse effects not only in healthy volunteers but in patients as well.

In conclusion, our findings identify miR-92a as a clinically relevant, disease-promoting miRNA that targets multiple pathways downstream of Foxo1 to modulate the regulatory and inflammatory T cell imbalance in EAE and MS. Our study provides functional insights into basic mechanisms of CNS autoimmunity mediated by miR-92a and identifies miR-92a silencing as a potential therapeutic approach for MS.

## Methods

Additional details are provided in the Supplemental Methods.

### Mice.

Inbred C57BL/6 (WT), *Mir92a^–/–^*, *Foxp3^gfp^*, *Il17a^gfp^*, *Ifng^yfp^*, B6.SJL-*Ptprc^a^* *Pepc^b^*/BoyJ (CD45.1), and B6.129S7-Rag1^tm1Mom^/J (*Rag1^–/–^*) mice were obtained from The Jackson Laboratory. *Mir92a^–/–^* mice had been backcrossed with mice on a C57BL/6 background for 10 or more generations before they were deposited with The Jackson Laboratory by the donating investigator. *Mir92a^–/–^* *Foxp3^gfp^* and *Mir92a^–/–^* *Il17a^gfp^* mice were generated in-house by crossing *Mir92a^–/–^* mice with *Foxp3^gfp^* and *Il17a^gfp^* mice, respectively. All mice were age matched (8–12 weeks old at the start of the experiments) and sex matched. Littermate controls were used when appropriate. Mice were maintained in specific pathogen–free animal facilities at the Harvard Institutes of Medicine at Harvard Medical School and the Hale Building for Transformative Medicine at Brigham and Women’s Hospital. Mice were maintained in 20°C–25°C, 50%–70% humidity and on a 12-hour light cycle, with the light phase beginning at 7 am and ending at 7 pm. Mice were housed with ad libitum access to food and water.

### Induction and evaluation of EAE.

EAE was induced via a well-established protocol (28). Eight- to 12-week-old mice were injected s.c. into both flanks with 100 μg MOG_35–55_ peptide (MEVGWYRSPFSRVVHLYRNGK) (Genemed Synthesis) dissolved in PBS and emulsified in CFA (BD) supplemented with 300 μg *M. tuberculosis* H37Ra (BD) (CFA/Mtb). Mice were also injected twice, i.p., with 150 ng pertussis toxin (PT) (List Biological Laboratories) administered on the day of immunization and 48 hours later. Clinical assessment of EAE was performed daily after disease induction according to the following criteria (28): 0, no disease; 1, tail paralysis; 2, loss of righting reflex and hind limb weakness; 2.5, hind limb partial paralysis; 3, complete hind limb paralysis; 3.5, complete hind limb paralysis and forelimb weakness; 4, complete hind limb paralysis and forelimb partial paralysis; 4.5, complete hind limb paralysis and forelimb paralysis; 5, moribund state. Mean clinical scores were calculated daily by adding the scores for individual mice and dividing them by the total number of mice in each group, including mice that did not develop signs of EAE. For histopathological studies, spinal cords were dissected, fixed in 10% formalin in PBS, and embedded in a single paraffin block. Sections (6–10 μm thick) were stained with H&E to evaluate immune cell infiltration and with Luxol Fast Blue (LFB) to evaluate demyelination.

### Induction of EAE via adoptive transfer of CD4^+^ T cells into Rag1^–/–^ mice.

Total CD4^+^ T cells were prepared from the spleens and inguinal LNs of WT and *Mir92a^–/–^* mice by positive selection via negative selection using a Mouse CD4^+^ T Cell Isolation Kit (Miltenyi Biotec; purity >95%) according to the manufacturer’s instructions. CD4^+^ T cells (2 × 10^7^) were injected i.v. into B6.129S7-Rag1^tm1Mom^/J (*Rag1^–/–^*) mice on a C57BL/6 background. Five days later, the recipient *Rag1^–/–^* mice were subjected to EAE and monitored for the condition.

### Mouse Th cell isolation and differentiation.

Total CD4^+^ T cells and naive CD4^+^ T cells from WT and *Mir92a^–/–^* mice were isolated from the spleens and inguinal LNs via negative selection using Mouse CD4^+^ T Cell Isolation Kits and Mouse Naive CD4^+^ T Cell Isolation Kits (Miltenyi Biotec), respectively, according to the manufacturer’s instructions. IMDM supplemented with 10% FBS, 1× penicillin/streptomycin, and 50 μM β-mercaptoethanol (Gibco, Thermo Fisher Scientific) was used for cell cultures. Naive CD4^+^ T cells were plated at 1 × 10^5^ cells/well on 96-well, flat-bottomed plates and activated with plate-bound anti–mouse CD3 (2 μg/mL, clone 145-2C11) and anti–mouse CD28 (2 μg/mL, clone 37.51) under Treg-polarizing conditions with recombinant human TGF-β (1 ng/mL); nonpathogenic Th17-polarizing conditions with recombinant human TGF-β (0.2 ng/mL), recombinant mouse IL-6 (30 ng/mL), and anti–mouse IFN-γ antibody (5 μg/mL, clone XMG1.2); pathogenic Th17-polarizing conditions with recombinant human TGF-β (0.2 ng/mL), recombinant mouse IL-1β (20 ng/mL), recombinant mouse IL-6 (30 ng/mL), recombinant mouse IL-23 (20 ng/mL), and anti-mouse IFN-γ antibody (5 μg/mL); or Th1-polarizing conditions with recombinant mouse IL-12 (20 ng/mL). Twenty-four to 48 hours after culturing, gene expression was analyzed by real-time qPCR. Four to 9 days after activation, cells were restimulated with PMA (50 ng/mL, MilliporeSigma) and ionomycin (1 μg/mL, MilliporeSigma) in the presence of Protein Transport Inhibitor (containing Monensin) (1:1000, BD Biosciences) for 4 hours at 37°C for intracellular cytokine analysis by flow cytometry. For Treg cultures with inflammatory cytokines, naive CD4^+^ T cells isolated from WT *Foxp3^gfp^* and *Mir92a^–/–^* *Foxp3^gfp^* mice were cultured under Treg-polarizing conditions for 3 days. FACS-sorted Foxp3-GFP^+^ Tregs were cultured with plate-bound anti–mouse CD3 and anti-mouse CD28, along with recombinant mouse IL-2 (10 ng/mL) and pathogenic Th17-polarizing cytokines (IL-1β/IL-6/IL-23) or Th1-polarizing cytokines (IL-12/IL-6) for 24, 48, and 72 hours. All recombinant cytokines and antibodies for culturing were purchased from R&D Systems and BioLegend, respectively.

### Treg suppression assays.

WT and *Mir92a^–/–^* mice were immunized for EAE, except without PT. On day 12 after immunization, total CD4^+^ T cells were isolated from draining inguinal LNs (dLNs) and spleens of these mice using Mouse CD4^+^ T Cell Isolation Kits (Miltenyi Biotec). Subsequently, CD4^+^Foxp3-GFP^+^ Tregs were FACS sorted from these total CD4^+^ T cells. Naive CD4^+^ T cells were isolated from B6.SJL-*Ptprc^a^* *Pepc^b^*/BoyJ (CD45.1) congenic WT mice through magnetic selection and then labeled with 1 μM CTV (Life Technologies, Thermo Fisher Scientific) according to the manufacturer’s instructions. APCs were isolated from CD45.1 congenic WT mice through negative magnetic selection using CD4 Microbeads and CD8 Microbeads (Miltenyi Biotec) to deplete T cells, according to the manufacturer’s instructions. CTV-labeled naive CD4^+^ T cells (Tresp cells) and APCs were plated at 5 × 10^4^ cells/well on 96-well, U-bottomed plates with soluble anti-CD3 (1 μg/mL) and varying numbers (1:1 to 1:8 of Tresp/Treg ratios) of WT or *Mir92a^–/–^* CD4^+^Foxp3-GFP^+^ Tregs for 3 days. Proliferation of Tresp cells was quantified by flow cytometry on the basis of CTV dilution. The percentage of suppression was calculated as: (frequency [percentage] of CTV-diluted Tresp cells cultured alone minus the percentage of CTV-diluted Tresp cells cultured with Tregs) divided by (percentage of CTV-diluted Tresp cells cultured alone), all multiplied by 100.

### HC and patient samples.

Cryopreserved PBMC samples from untreated patients with RRMS and age- and sex-matched HC donors were obtained from the Comprehensive Longitudinal Investigation of Multiple Sclerosis at Brigham and Women’s Hospital (CLIMB) study. CLIMB maintains an ongoing longitudinal cohort study that follows more than 2000 patients with clinical examinations. Patients in this study are diagnosed with MS as defined by the 2017 revisions to the McDonald criteria (34). Patients with RRMS selected for this study had not received treatment with steroids in the prior month or other disease-modifying therapies in the preceding 3 months.

### Human Th cell isolation and differentiation.

PBMCs were isolated from whole blood obtained from HC donors by Ficoll (Pharmacia LKB Biotechnology) gradient centrifugation. Total and naive CD4^+^ T cells from PBMCs of HC donors and patients with RRMS were isolated through magnetic selection using Human CD4^+^ T Cell Isolation Kits and Human Naive CD4^+^ T Cell Isolation Kits (both from Miltenyi Biotec), respectively, according to the manufacturer’s instructions. Serum-free X-VIVO 15 Medium (Lonza) was used for cell cultures. Naive CD4^+^ T cells from HCs and patients with MS were plated at 1 × 10^5^ cells/well on 96-well, U-bottomed plates and cultured with plate-bound anti–human CD3 (5 μg/mL, clone UCHT1) and soluble anti–human CD28 (1 μg/mL, clone CD28.2) under Treg-polarizing conditions with recombinant human TGF-β (1 ng/mL) and recombinant human IL-2 (10 ng/mL); Th17-polarizing conditions with TGF-β (1 ng/mL), recombinant human IL-1β (12.5 ng/mL), recombinant human IL-6 (25 ng/mL), recombinant human IL-23 (25 ng/mL), anti–human IFN-γ antibody (1 μg/mL, clone B27), and anti–human IL-4 antibody (1 μg/mL, clone MP4-25D2); or Th1-polarizing conditions with recombinant human IL-12 (20 ng/mL). Twenty-four to 96 hours after culturing, transcription factor expression was analyzed by real-time qPCR. Four to 9 days after activation, cells were restimulated with PMA and ionomycin in the presence of GolgiStop for 4 hours for intracellular cytokine analysis by flow cytometry. All recombinant cytokines and antibodies for culturing were purchased from R&D Systems and BD Biosciences, respectively.

### miR-92a inhibitor treatments.

LNA-modified miR-92a inhibitor (miRCURY LNA mmu-miR-92a-3p Power Inhibitor) and LNA control inhibitor (Negative Control A) were purchased from QIAGEN and reconstituted in sterile nuclease-free water at a concentration of 10 μM. According to the manufacturer, the control inhibitor has no hits of greater than 70% homology to any sequence in any organism in the NCBI and miRbase databases, minimizing the chance it targets endogenous RNAs to produce any physiological impact. For in vitro treatment, 10 nM miR-92a inhibitor or control inhibitor was added to splenocytes or mouse or human total CD4^+^ T cells, followed by qPCR after 48 hours. For mouse Th cell polarizations, 10–20 nM miR-92a inhibitor or the control inhibitor was added to naive CD4^+^ T cells and left for the entire duration of culturing, followed by intracellular staining and flow cytometric analyses. For in vivo treatment, the miR-92a inhibitor (miRCURY LNA mmu-miR-92a-3p Custom Inhibitor large scale) and the control inhibitor (negative control A) (25 mg/kg) were diluted in PBS, and then administered i.p. in a volume of 150 μL on days 3, 5, 7, 9, and 11 after MOG_35–55_/CFA/Mtb immunization. The treated mice were monitored for EAE. Some treated mice were sacrificed at the onset of EAE to examine Th cells in the spleens and dLNs by flow cytometry. For HC and MS Th polarizations, 10–20 nM miR-92a inhibitor or control inhibitor was added to human naive CD4^+^ T cells and left for the entire culture duration, followed by intracellular staining and flow cytometric analyses. The inhibitor sequences were as follows: miRCURY LNA mmu-miR-92a-3p Power Inhibitor, AGGCCGGGACAAGTGCAAT; miRCURY LNA mmu-miR-92a-3p Custom Inhibitor large scale, CGGGACAAGTGCAAT; negative control A, TAACACGTCTATACGCCCA; and miRCURY LNA hsa-miR-92a-3p Power Inhibitor, AGGCCCGGGACAAGTGCAAT.

### Data analysis.

BD FlowJo, version 10.7.2, was used to analyze flow cytometric data and prepare the figures. A Leica Biosystems Aperio ImageScope 12.4.3 was used to prepare the microscopy images. GraphPad Prism 9.1.2 (GraphPad Software) was used to generate the figures and conduct statistical analyses. Adobe Illustrator, version 24.3, was used to design the figures.

### Statistics.

For T cell studies involving HCs and patients with MS, statistical analyses were performed using the Wilcoxon signed-rank test or the Mann-Whitney *U* test, followed by normality tests with the Shapiro-Wilk test or the Kolmogorov-Smirnov test using GraphPad Prism 9.1.2. For animal studies, statistical analyses were performed with a Mann-Whitney *U* test, an unpaired, 2-tailed Student’s *t* test, or 1-way ANOVA with appropriate corrections for multiple comparisons (Šidák’s or Dunnet’s), using GraphPad Prism 9.1.2. A *P* value of less than 0.05 was considered statistically significant. Data are presented as the mean ± SEM.

### Study approval.

All animal procedures were performed in accordance with guidelines from the IACUC of Harvard Medical School and Brigham and Women’s Hospital. All experiments involving HC donor samples and samples from patients with MS were reviewed and approved by the IRB of Brigham and Women’s Hospital. Signed informed consent was received from all participants. 

## Author contributions

MF and RR designed and performed experiments and analyzed data. AKA performed luciferase assays and ChIP studies and analyzed data. LPG, RK, SHK, GG, RK, VB, A Patel, SS, DH, BH, A Paul, and RG performed experiments. TC and HLW selected the patients with MS and HC donors and arranged for sample collections. MF and GM prepared the manuscript, with participation from RR and LPG. GM designed and supervised the overall study.

## Supplementary Material

Supplemental data

## Version 1 03/17/2022

In-Press Preview

## Version 2 05/16/2022

Electronic publication
