# Supplementary material for: microRNA-92a promotes CNS autoimmunity by modulating the regulatory and inflammatory T cell balance
Source: J Clin Invest. 2022 May 16;132(10):e155693. doi: 10.1172/JCI155693 (PMC9106347; doi:10.1172/JCI155693)
Supplement: Supplemental data [file jci-132-155693-s021.pdf]

Supplemental Information

MicroRNA-92a promotes CNS autoimmunity by modulating the regulatory and inflammatory T cell balance

Mai Fujiwara[1,*], Radhika Raheja[1,*], Lucien P. Garo[1,2], Amrendra K. Ajay[3], Ryoko Kadowaki-Saga[1], Sukrut H. Karandikar[1], Galina Gabriely[1], Rajesh Krishnan[1], Vanessa Beynon[1], Anu Paul[1], Amee Patel[1], Shrishti Saxena[1], Dan Hu[1], Brian Healy[1], Tanuja Chitnis[1], Roopali Gandhi[1], Howard L. Weiner[1], and Gopal Murugaiyan[1,*]

[1]Ann Romney Center for Neurologic Diseases, Brigham and Women's Hospital and Harvard Medical School, 60 Fenwood Road, Boston, MA, 02115, USA.

[2]Pulmonary Center, Department of Medicine, Boston University School of Medicine, Boston, MA, 02118, USA.

[3]Renal Division, Department of Medicine, Brigham and Women's Hospital and Harvard Medical School, Boston, MA, 02115, USA.

Correspondence: mgopal@rics.bwh.harvard.edu

Supplemental Methods

Preparation and evaluation of immune cells from the periphery and the CNS

For EAE onset, spleens and draining inguinal lymph nodes (dLNs) were harvested, red blood cells (RBCs) were lysed, then remaining cells were passed through a 70 μm cell strainer. For assessing MOG_{35-55} recall responses, RBC-lysed splenocytes and dLN cells were cultured in the presence of 25 μg/ml MOG_{35-55} for 3 d. For EAE peak, animals were perfused with cold PBS. Brains and spinal cords were dissected and incubated in collagenase D (2.5 mg/ml, Sigma-Aldrich) and DNase (1 mg/ml, Sigma-Aldrich) for 40 min at 37°C. Single-cell suspensions were prepared by passing them through a 70 μm cell strainer. Cells were washed in Iscove's Dulbecco's modified medium (IMDM) (Gibco), and mononuclear cells were isolated using a discontinuous (37% and 70%) Percoll gradient (Fisher Scientific). Cells were washed twice in IMDM. Either the splenocytes ,dLN cells, or cells isolated from the CNS were cultured with media containing phorbol myristate acetate (PMA) (50 ng/ml, Sigma-Aldrich) and ionomycin (1 μg/ml, Sigma-Aldrich) in the presence of Protein Transport Inhibitor (containing Monensin) (1:1,000, BD Biosciences) for 4 h at 37°C for intracellular cytokine analysis by flow cytometry.

Effector $CD4^+$ T cell-mediated adoptive transfer of EAE in C57BL/6 mice

8-12-week-old WT and *Mir92a*$^{-/-}$ mice were injected s.c. with 150 μg $MOG_{35–55}$ and 300 μg CFA/Mtb using a previously described protocol (1). On day 10, dLNs were harvested from these immunized WT and *Mir92a*$^{-/-}$ mice and cultured with 30 μg/ml MOG_{35-55}, 6 ng/ml recombinant mouse IL-23 (R&D Systems), and 3 ng/ml recombinant mouse IL-12 (R&D Systems), for 3 d. At the end of the culture, cells were washed and $CD4^+$ T cells were purified by negative selection using a Mouse $CD4^+$ T Cell Isolation Kit (Miltenyi Biotec) according to the manufacturer's instructions. $5x10^6$ $CD4^+$ T cells/mouse were injected i.p. into recipient naïve C57BL/6 WT mice. Recipient WT mice were also injected i.p. with 75 ng PT administered on the day of cell transfer and 48 h later, then monitored for EAE.

Co-culture assays

$CD11c^+$ DCs were isolated from the spleens and LNs of naïve WT and *Mir92a*$^{-/-}$ mice via magnetic selection using CD11c Microbeads (Miltenyi Biotec). Naïve $CD4^+CD62L^{hi}CD44^{lo}$ T cells were isolated from the spleens

and LNs of *Ifng*^{yfp}, *Foxp3*^{gfp}, or *Il17a*^{gfp} mice via magnetic selection using a Mouse Naïve CD4$^+$ T Cell Isolation Kit (Miltenyi Biotec) according to the manufacturer's instructions. Using a previously described protocol (2), naïve CD4$^+$ T cells were then co-cultured with DCs at a 1:1 DC:CD4$^+$ T cell ratio with soluble anti-CD3 (1 μg/ml) and anti-CD28 (1 μg/ml) in the presence of Th1-, Treg- or Th17-polarizing cytokines (refer to mouse T helper differentiation in **Methods**) (2) in U-bottom 96-well plates for 3-5 d, then analyzed by flow cytometry.

Flow cytometry

Briefly, mouse and human immune cells were stained with Live/Dead-aqua (Life Technologies) prior to fixation. For Foxp3 staining alone or Foxp3 and intracellular cytokine co-staining, cells were fixed and permeabilized using the Transcription Factor Staining Kit (Life Technologies) according to the manufacturer's instructions. Cells were stained with fluorochrome-conjugated antibodies: anti-mouse CD4-Brilliant Violet 421 (clone GK1.5,) or anti-human CD4-Pacific Blue (clone RPA-T4), anti-mouse IL-17A-APC (clone TC11-18H10.1), anti-mouse IFN-γ-PE-Cy7 (clone XMG1.2), and anti-rat/mouse/human Foxp3-AlexaFluor 647 (clone 150D).

For Foxo1 staining, cells were fixed with 2% paraformaldehyde, followed by fixation with 100% methanol. Cells were then stained with the following: anti-human CD4-APC (clone RPA-T4) and anti-mouse/human Foxo1-PE (clone C29H4, Cell Signaling Technology).

For intracellular cytokine staining, cells were fixed and permeabilized using the BD Cytoperm/Cytofix Kit (BD Biosciences) according to the manufacturer's instructions. Total splenocytes or LN cells or human PBMCs were then incubated with either mouse or human FcR block (Miltenyi Biotec) according to the manufacturer's instructions. Cells were then stained with the following: anti-mouse CD4-Brilliant Violet 421, anti-mouse IL-17A-APC, anti-mouse IFN-γ-PE-Cy7, anti-mouse GM-CSF-PE (clone MP1-22E9), anti-mouse IL-1R-PE (clone JAMA-147), and anti-mouse IL-23R-PE (clone 12B2B64); or anti-human CD4-Pacific Blue, anti-human IL-17A-AlexaFluor647 (clone N49-653, BD Biosciences), anti-human IFN-γ-PE-Cy7 (clone 4S.B3), anti-human IL-1R-PE (polyclonal, R&D Systems), and anti-human IL-1R-PE (clone 218213, R&D Systems).

For surface staining, live cells were stained with 7-AAD (BioLegend), anti-mouse CD11c-Brilliant Violet 421 or Pacific Blue (clone N418), anti-mouse MHC-II-PE or APC-Cy7 (clone M5/114.15.2), anti-mouse CD11b-FITC (clone M1/70), anti-mouse F4/80-APC (clone BM8), anti-mouse CD19-APC (clone 1D3), anti-mouse B220-FITC (clone RA3-6B2), anti-mouse CD4-PE or APC (clone GK1.5), anti-mouse CD8a-APC (clone 53-6.7), anti-

mouse NK1.1-FITC (clone PK136), anti-mouse CD49b-APC (clone DX5), anti-mouse CD80-FITC (clone 16-10A1), anti-mouse CD86-PE (clone GL-1), anti-mouse CD45.1-PE (clone A20). All cells were washed and resuspended in 2% FBS-containing PBS to be acquired on the Beckton Dickinson (BD) LSR II.

For Treg-polarizing conditions, WT *Foxp3*^{gfp} and *Mir92a*$^{-/-}$*Foxp3*^{gfp} mice were used, and levels of Foxp3-GFP were measured. For non-pathogenic Th17-polarizing conditions, WT *Il17a*^{gfp} and *Mir92a*$^{-/-}$*Il17a*^{gfp} mice were used, and levels of IL-17A-GFP were measured. For FACS of CD4^{+} T cells from the CNS of WT mice with EAE, mononuclear cells from the CNS were stained with 7-AAD (BioLegend), anti-mouse CD19-PerCP (clone 6D5), anti-mouse NK1.1-PerCP (clone PK136), anti-mouse Ly6G-PerCP (clone 1A8), anti-mouse CD45-APC (clone 30-F11), anti-mouse CD3-Alexa Fluor 700 (clone 17A2), and anti-mouse CD4-PE, then sorted based on single 7-AAD^{-}Dump^{-}(CD19^{-}NK1.1^{-}Ly6G^{-}) CD45^{+}CD3^{+}CD4^{+} cells. For FACS of mouse Tregs generated in vitro, cultured CD4^{+} T cells were stained with 7-AAD and anti-mouse CD4-APC, then sorted based on single 7AAD^{-} CD4^{+}Foxp3-GFP^{+} cells. For FACS of mouse Tregs from LN cells and splenocytes from mice with EAE, cells were enriched for CD4^{+} T cells using Mouse CD4 Microbeads (Miltenyi Biotec) and sorted as above. All samples were filtered through 70 μm nylon prior to being sorted using a 70 μm nozzle on BD Aria IIu SORP cell sorters.

For all flow cytometric analyses, gates were set using Fluorescence Minus One (FMO) controls (3, 4). For flow cytometric analyses based on histograms, geometric Mean Fluorescence Intensity values (MFIs) were used. MFI values of FMOs were subtracted from all corresponding MFI values where indicated in figure legends (5-8), or represented as fold change (FC) values where indicated in figure legends. All flow cytometry antibodies, unless noted otherwise, were purchased from BioLegend.

RNA isolation, cDNA synthesis, and quantitative RT-PCR

Total RNA was isolated from cell pellets using Norgen Total RNA Purification Micro Kit (Norgen Biotek, Canada) according to the manufacturer's instructions. Complementary DNA (cDNA) synthesis was performed for each RNA sample from 50-100 ng of total RNA using a High-Capacity cDNA Synthesis Kit with RNase Inhibitor (Life Technologies). cDNA was amplified using sequence-specific probes and Fast Advanced Real-Time PCR Mix (Applied Biosystems, Thermo Fisher Scientific) on the QuantStudio Flex 7 Real-Time PCR system (Thermo Fisher). The *Gapdh* gene was used as an endogenous control to normalize for differences in the amount of total RNA in each sample. Normalized qRT-PCR data are represented as fold change (FC) relative to WT or control

inhibitor conditions. The probes used are identified by the following Applied Biosystems assay numbers: *Rorc*, Mm01261019_m1; *Il23r*, Mm00519942_m1; *Il23a*, Mm01160011_m1; *Il6*, Mm99999064_m1; *Il1r1*, Mm00434237_m1; *Il1b*, Mm01336189_m1; *Tgfb1*, Mm01178820_m1; *Il12a*, Mm00434169_m1; *Foxo1*, Mm00490672_m1; *Rel*, Mm01239658_m1; *Ikzf4*, Mm01133256_m1; *Stat5b*, Mm00839889_m1; *Nfatc2*, Mm01240677_m1; *Stat5a*, Mm00839861_m1; *Bach2*, Mm00464379_m1; *Ets1*, Mm01175819_m1; *Ikzf1*, Mm01187877_m1; *Socs3*, Mm00545913_m1; *Ikzf2*, Mm00496108_m1; *Socs1*, Mm00782550_s1; *Ikzf3*, Mm01306721_m1; *Satb1*, Mm01268940_m1; *Maf*, Mm02581355_s1; *Gapdh*, Mm99999915_g1; *RORC*, Hs01076112_m1; *GAPDH*, Hs02786624_g1.

Analyses of miR-92a expression

For analyses of miR-92a expression in mice and HC and MS patient CD4$^+$ T cells, qRT-PCR was performed using TaqMan MicroRNA Assays (Applied Biosystems, Thermo Fisher Scientific). cDNA synthesis was performed for each RNA sample from 15-30 ng of total RNA using a High-Capacity cDNA Synthesis Kit with RNase Inhibitor (Life Technologies). cDNA was amplified using sequence-specific probes and Fast Advanced Real-Time PCR Mix (Applied Biosystems, Thermo Fisher Scientific) on the QuantStudio Flex 7 Real-Time PCR system (Thermo Fisher). Relative expression was calculated using Ct values and normalized to housekeeping genes (snoRNA135 for mice and U6 snRNA for humans). Normalized qRT-PCR data are represented as FC relative to WT or control inhibitor conditions. The probes used are identified by the following Applied Biosystems assay numbers: mmu-miR-92a-3p (mouse), 000430; hsa-miR-92a-3p (human), 000431; snoRNA135, 001230 (for both mouse and human); U6 snRNA, 001973 (for both mouse and human).

Luciferase assays

To generate Firefly luciferase reporters for Foxo1, fragments of *Foxo1* 3'UTR containing corresponding putative miR-92a binding sites were cloned into the *NotI* and *XhoI* sites of psiCHECK-2 vector (Promega), downstream of *Renilla* luciferase. HEK293T cells grown in DMEM supplemented with 10% FBS were co-transfected with either psiCHECK-2 vector containing *Foxo1* 3'UTR fragments or empty vector in the presence of mirVana mmu-miR-92a-3p mimic oligo (Thermo Fisher), miRCURY locked nucleic acid (LNA) mmu-miR-92a-3p Power Inhibitor (Qiagen), or the LNA control (Negative Control A). After 48 h, luciferase activities were measured with the Dual-

Glo Luciferase Assay System (Promega), and *Renilla* luciferase activity was normalized to *Firefly* luciferase activity. Sequences for mirVana mmu-miR-92a-3p mimic oligo: UAUUGCACUUGUCCCGGCCUG; miRCURY LNA mmu-miR-92a-3p Power Inhibitor: AGGCCGGGACAAGTGCAAT; Negative Control A: TAACACGTCTATACGCCCA.

SiRNA transfection

Foxo1 siRNA (*Silencer*™ Select Pre-Designed siRNA; ID s80621) and a negative control (*Silencer*™ Select Negative Control #2) were purchased from Life Technologies. Briefly, mouse naïve CD4$^+$ T cells were cultured under either Treg-, non-pathogenic Th17-, or pathogenic Th17-polarizing conditions without any penicillin/streptomycin. After 24 h, *Foxo1* siRNA and negative control siRNA were mixed with Trans-IT TKO transfection reagent (Mirus), then added to cells at 25 nM. After 16 h, media containing siRNAs were replaced with fresh media containing Th polarizing cytokines. Cells were incubated at 37°C for 3 d (Treg) or 4 d (Th17), and analyzed by flow cytometry. *Foxo1* siRNA sequence sense (s): GCACCGACUUUAUGAGCAAtt, antisense (as): UUGCUCAUAAAGUCGGUGCtg.

Chromatin immunoprecipitation (ChIP)

ChIP was performed using the Magna ChIP G Kit per the manufacturer's instructions (EMD Millipore) (2). $3x10^6$ splenic naïve CD4$^+$ T cells isolated from WT and *Mir92a*$^{-/-}$ mice were stimulated with 2 μg/ml plate-bound anti-CD3 and soluble anti-CD28 for 24 h in the presence of Treg-, nonpathogenic Th17-, or pathogenic Th17-polarizing cytokines. DNA was cross-linked with protein by adding 1% formaldehyde to the wells. Sonication was performed to obtain DNA fragments ranging from 200-1000 bp. Samples were spun at 10,000xg at 4°C for 10 min, and 500 μg protein was used in 500 ml volume for IP with 2 μg anti-Foxo1, anti-RORγt, or normal rabbit IgG (negative control). 5 μg (1%) protein from each sample was frozen in 80°C as input. Protein G magnetic bead–antibody/chromatin complexes were separated by magnetic rack and washed with 500 ml low-salt immune complex wash buffer, high-salt immune complex wash buffer, LiCl immune complex wash buffer, and tris-ethylenediaminetetraacetic acid buffer. 100 μl ChIP elution buffer/proteinase K was added in IP, as well as input samples, and incubated at 62°C for 2 h with shaking. Proteinase K was denatured at 95°C for 10 min. For IP samples, protein G magnetic beads were separated by a magnetic separator rack, and supernatant was used

for DNA purification. 5 μl purified DNA was used for real time PCR using promoter-target specific primers of *Foxp3* CNS1, *Foxp3* CNS3, and promoter loci of *Il17a*, *Il1r1*, *Il23r*, and *Gapdh* (normalizer). Primer sequences: *Foxp3* CNS1 s: 5' CCCTGCAATTATCAGCACAC 3', as: 5' TGTGGGAAACTGCCACATTA 3'; *Foxp3* CNS3 s: 5' ATCTGGCCAAGTTCAGGTTG 3', as: 5' GGCGTTCCTGTTTGACTGTT 3'; *Il17a* promoter s: 5' AACTTCTGC CCTTCCCATCT 3', as: 5' GTTTGCGCGTCCTGATCAGC 3'; *Il1r1* promoter region 1 s: 5' AAGGGTGCAGTCTC CTAACG 3'; as: 5' TCTCTCCTTGCCTAGGTCTGT 3'; *Il1r1* promoter region 2 s: 5' AGCGCCCTTTTACCGTAACT 3', s: 5' CCCCTCAGCACACTTTCA GA 3'; *Il23r* promoter region 1 s: 5' TAGGGTTGGGAAATGAGGCTGACA 3', as: 5' AAGGAGTGCAAGAGGAGCATTGGA 3'; *Il23r* promoter region 2 s: 5' TCTCTCGTACTGCCAATTGCACCT 3', as: 5' AGGCAGCACAAGCTGTAGAATTGC 3'.

Treg depletion with anti-CD25 antibody during EAE

WT and *Mir92a*$^{-/-}$ mice were immunized with $MOG_{35\text{-}55}$ and CFA/Mtb and administered i.p. with 250 μg anti-CD25 monoclonal antibody (clone PC-61.5.3, Bio X Cell) or rat IgG_1 isotype control (clone HRPN, Bio X Cell) on days 0, 3, 6, and 9 post-immunization. The treated mice were sacrificed on day 12 post-immunization to examine the T helper cells in the spleens and dLNs by flow cytometry.

References

1. Murugaiyan G, et al. MicroRNA-21 promotes Th17 differentiation and mediates experimental autoimmune encephalomyelitis. *J Clin Invest.* 2015;125(3):1069-80.
2. Garo LP, et al. Smad7 Controls Immunoregulatory PDL2/1-PD1 Signaling in Intestinal Inflammation and Autoimmunity. *Cell Rep.* 2019;28(13):3353-66 e5.
3. Baumgarth N, and Roederer M. A practical approach to multicolor flow cytometry for immunophenotyping. *J Immunol Methods.* 2000;243(1-2):77-97.
4. Perfetto SP, et al. Seventeen-colour flow cytometry: unravelling the immune system. *Nat Rev Immunol.* 2004;4(8):648-55.
5. Akkaya B, et al. A Simple, Versatile Antibody-Based Barcoding Method for Flow Cytometry. *J Immunol.* 2016;197(5):2027-38.

6. Akkaya M, et al. Toll-like receptor 9 antagonizes antibody affinity maturation. *Nat Immunol.* 2018;19(3):255-66.

7. Cassado Ados A, et al. Cellular renewal and improvement of local cell effector activity in peritoneal cavity in response to infectious stimuli. *PLoS One.* 2011;6(7):e22141.

8. Skopelja-Gardner S, et al. Acute skin exposure to ultraviolet light triggers neutrophil-mediated kidney inflammation. *Proc Natl Acad Sci U S A.* 2021;118(3).

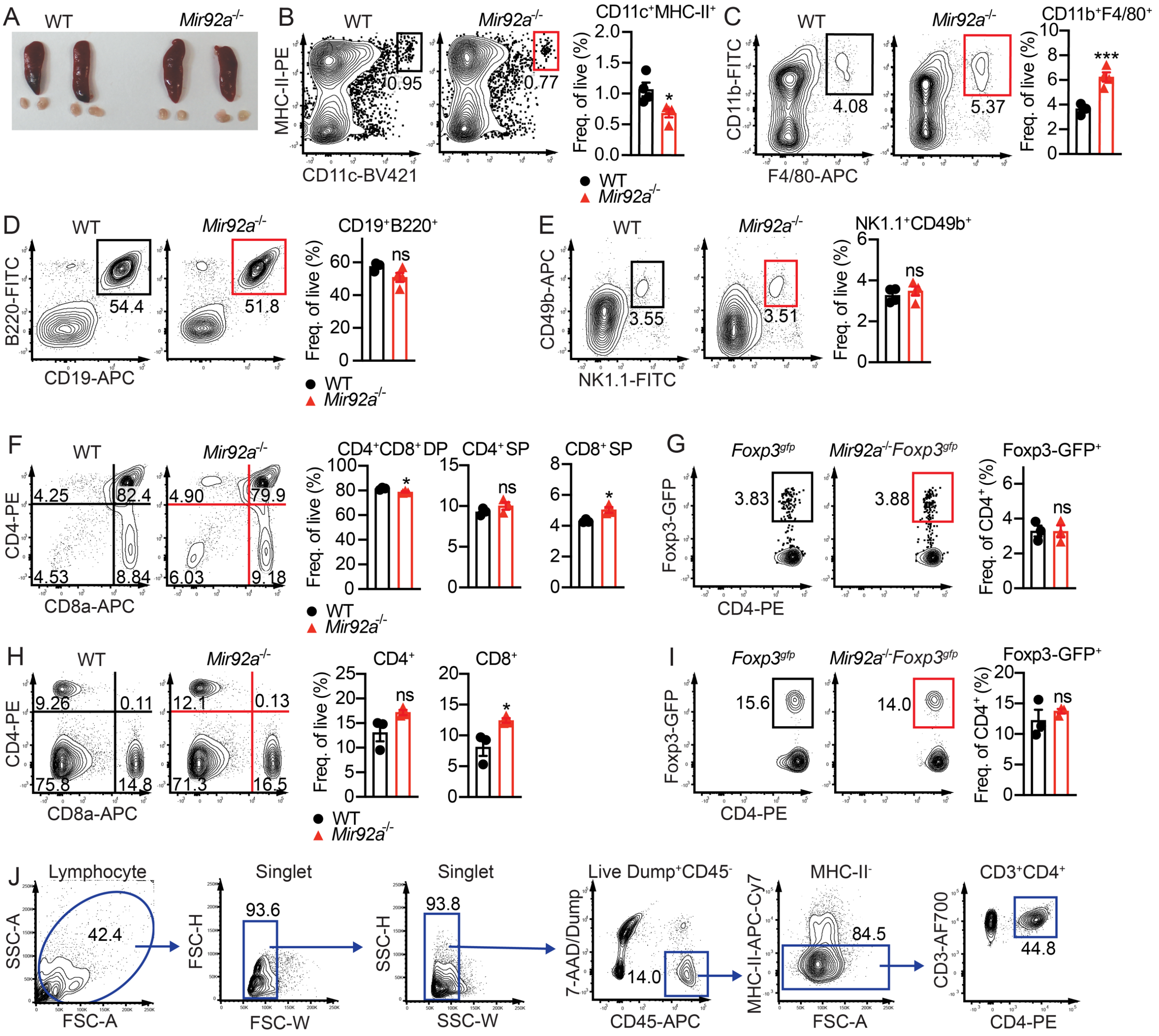
A
WT
Mir92a-/-
B
WT
Mir92a-/-
MHC-II-PE
CD11c-BV421
0.95
0.77
CD11c+MHC-II+
Freq. of live (%)
2.0
1.5
1.0
0.5
0.0
*
WT
Mir92a-/-
C
WT
Mir92a-/-
CD11b-FITC
F4/80-APC
4.08
5.37
CD11b+F4/80+
Freq. of live (%)
10
8
6
4
2
0
***
D
WT
Mir92a-/-
B220-FITC
CD19-APC
54.4
51.8
CD19+B220+
Freq. of live (%)
60
40
20
0
ns
WT
Mir92a-/-
E
WT
Mir92a-/-
CD49b-APC
NK1.1-FITC
3.55
3.51
NK1.1+CD49b+
Freq. of live (%)
6
4
2
0
ns
F
WT
Mir92a-/-
CD4-PE
CD8a-APC
4.25
82.4
4.53
8.84
4.90
79.9
6.03
9.18
CD4+CD8+ DP
CD4+ SP
CD8+ SP
Freq. of live (%)
100
80
60
40
20
0
15
10
5
0
8
6
4
2
0
*
ns
*
WT
Mir92a-/-
G
Foxp3gfp
Mir92a-/-Foxp3gfp
Foxp3-GFP
CD4-PE
3.83
3.88
Foxp3-GFP+
Freq. of CD4+ (%)
6
4
2
0
ns
H
WT
Mir92a-/-
CD4-PE
CD8a-APC
9.26
0.11
75.8
14.8
12.1
0.13
71.3
16.5
CD4+
CD8+
Freq. of live (%)
25
20
15
10
5
0
20
15
10
5
0
ns
*
WT
Mir92a-/-
I
Foxp3gfp
Mir92a-/-Foxp3gfp
Foxp3-GFP
CD4-PE
15.6
14.0
Foxp3-GFP+
Freq. of CD4+ (%)
20
15
10
5
0
ns
J
Lymphocyte
SSC-A
FSC-A
42.4
Singlet
FSC-H
FSC-W
93.6
Singlet
SSC-H
SSC-W
93.8
Live Dump+CD45-
7-AAD/Dump
CD45-APC
14.0
MHC-II-
MHC-II-APC-Cy7
FSC-A
84.5
CD3+CD4+
CD3-AF700
CD4-PE
44.8

Supplemental Figure 1. Immunophenotyping of naïve *Mir92a$^{-/-}$* mice.

(A) Images of spleens and lymph nodes from 8-week-old naïve WT and *Mir92a$^{-/-}$* mice (n=2). (B-E) Representative flow cytometric plots and frequencies of CD11c$^+$MHC-II$^+$ DCs (B), CD11b$^+$F4/80$^+$ macrophages (C), B220$^+$CD19$^+$ B cells (D), and NK1.1$^+$CD49b$^+$ NK cells (E), in the spleens of naïve WT and *Mir92a$^{-/-}$* mice (n=4). (F) Representative flow cytometric plots and frequencies of CD4$^+$CD8$^+$ double positive (DP), CD4$^+$ single positive (SP), and CD8$^+$ SP T cells in the thymi of naïve WT and *Mir92a$^{-/-}$* mice (n=3). (G) Representative flow cytometric plots and frequencies of Foxp3$^+$ CD4$^+$ T cells in the thymi of naïve WT and *Mir92a$^{-/-}$* mice (n=3). (H) Representative flow cytometric plots and frequencies of CD4$^+$ T cells and CD8$^+$ T cells and in the spleens of naïve WT and *Mir92a$^{-/-}$* mice (n=3). (I) Representative flow cytometric plots and frequencies of Foxp3$^+$ CD4$^+$ T cells in the spleens of naïve WT and *Mir92a$^{-/-}$* mice (n=3). (J) FACS gating strategy for CD4$^+$ T cells isolated from the CNS of WT mice at peak disease during EAE: Lymphocyte, singlet, 7-AAD$^-$Dump(CD19/NK1.1/Ly6G)$^-$CD45$^+$, MHC-II$^-$, CD3$^+$CD4$^+$. Data representative of ≥2 independent experiments. Mean±SEM. ns=not statistically significant, *p<0.05, ***p<0.001 by unpaired t-test (B-I).

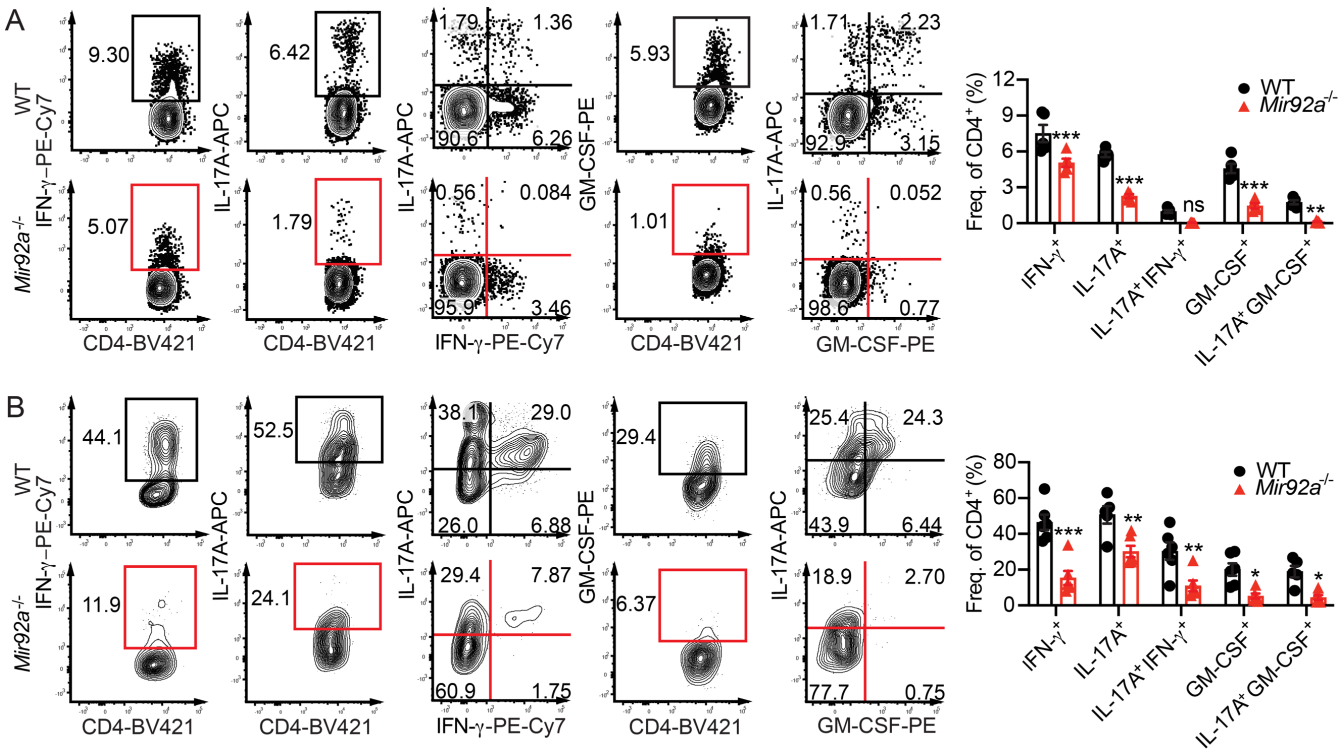

Supplemental Figure 2. MiR-92a deficiency leads to lower Th1 and Th17 cytokine expression during EAE. (A, B) Representative flow cytometric plots (left) and frequencies (right) of IFN-γ$^+$, IL-17A$^+$, IFN-γ$^+$IL-17A$^+$, GM-CSF$^+$, and IL-17A$^+$GM-CSF$^+$ CD4$^+$ T cells in the spleen of WT and *Mir92a*$^{-/-}$ mice at EAE onset (n=5) ex vivo (A), and after 72 h restimulation with MOG$_{35-55}$ (B). Data representative of ≥2 independent experiments. Mean±SEM. ns=not statistically significant, *p<0.05, **p<0.01, ***p<0.001, by one-way ANOVA with Sidak's multiple comparison test between WT and *Mir92a*$^{-/-}$ within each condition.

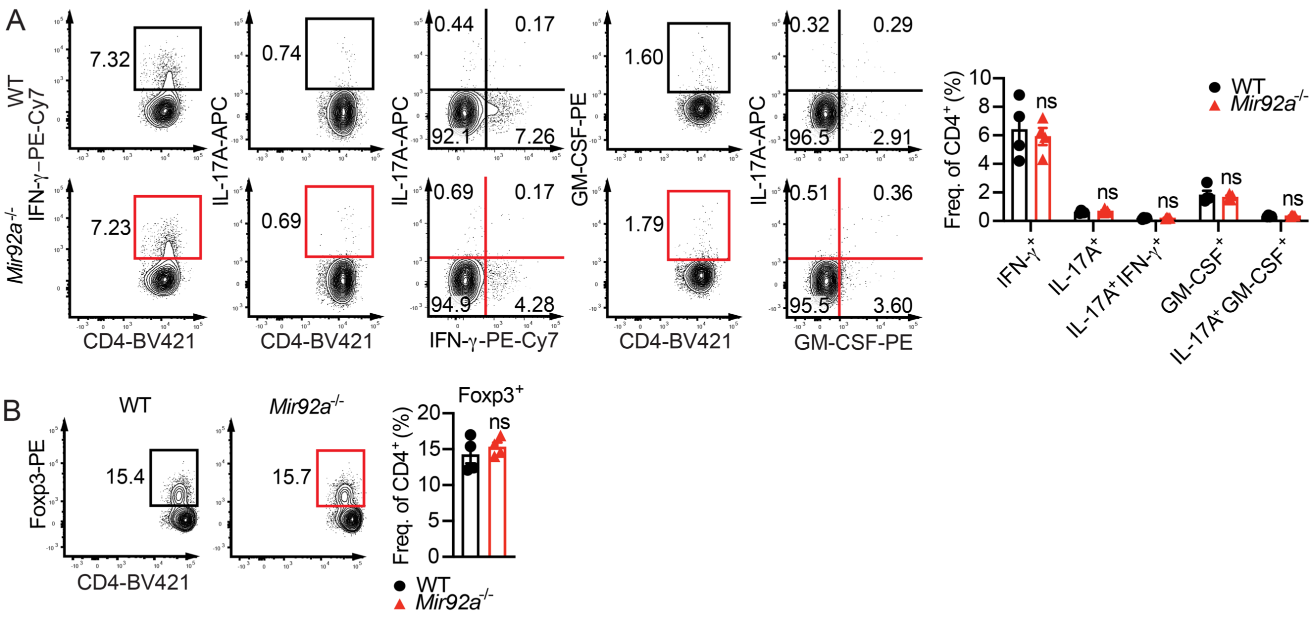

Supplemental Figure 3. MiR-92a deficiency does not alter inflammatory and regulatory CD4$^+$ T cells in response to CFA/Mtb immunization without MOG$_{35-55}$ peptide.

(A, B) Representative flow cytometric plots (left) and frequencies (right) of IFN-γ$^+$, IL-17A$^+$, IFN-γ$^+$IL-17A$^+$, GM-CSF$^+$, and IL-17A$^+$GM-CSF$^+$ (A), and Foxp3$^+$ (B), CD4$^+$ T cells in the spleens of WT and *Mir92a*$^{-/-}$ mice immunized with CFA/Mtb (n=4). Data representative of 2 independent experiments. Mean±SEM. ns=not statistically significant, by one-way ANOVA with Sidak's multiple comparison test between WT and *Mir92a*$^{-/-}$ within each condition (A), or unpaired t-test (B).

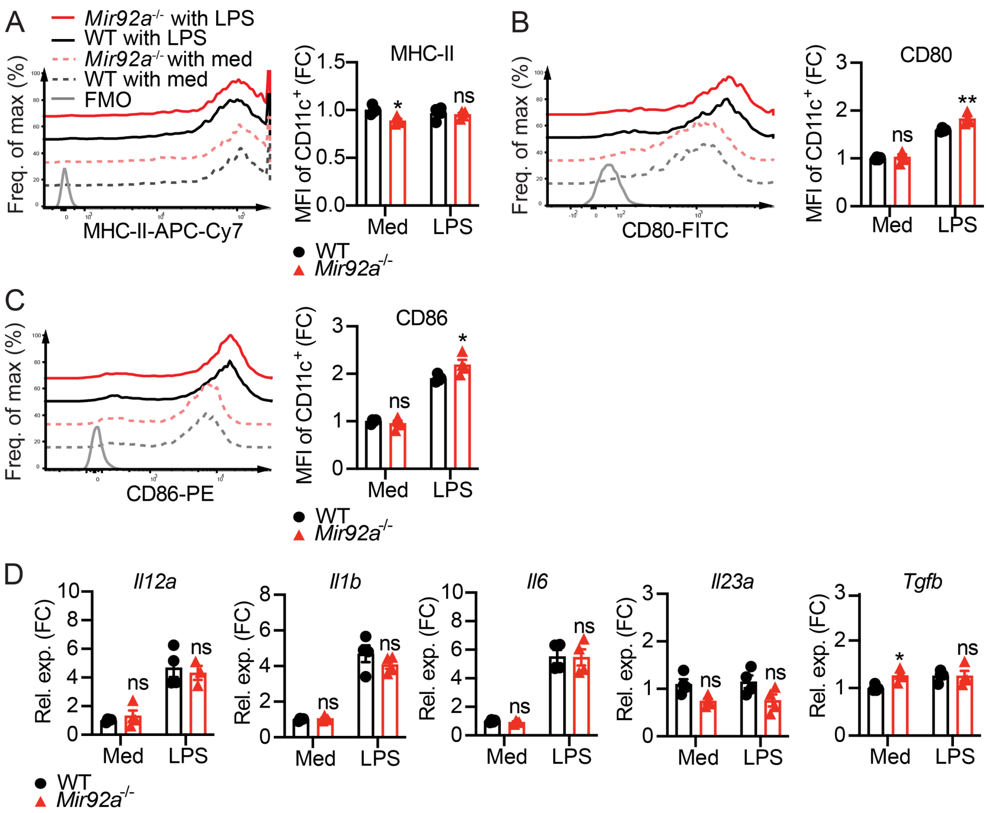

Supplemental Figure 4. MiR-92a deficiency does not affect DC phenotype and function.

(A-C) Representative flow cytometric histograms and MFIs of MHC-II (A), CD80 (B), and CD86 (C) in WT and *Mir92a*$^{-/-}$ CD11c^{+} DCs stimulated with or without LPS (n=4). (D) qPCR analyses of T helper polarizing cytokines in WT and *Mir92a*$^{-/-}$ CD11c^{+} DCs from A-C (n=3-4). MFIs represented as FC relative to WT media condition. qPCR data (normalized to *Gapdh*) represented as FC relative to WT media condition. Data representative of ≥2 independent experiments. Mean±SEM. ns=not statistically significant, *p<0.05, **p<0.01 by one-way ANOVA with Sidak's multiple comparison test between WT and *Mir92a*$^{-/-}$ within each condition.

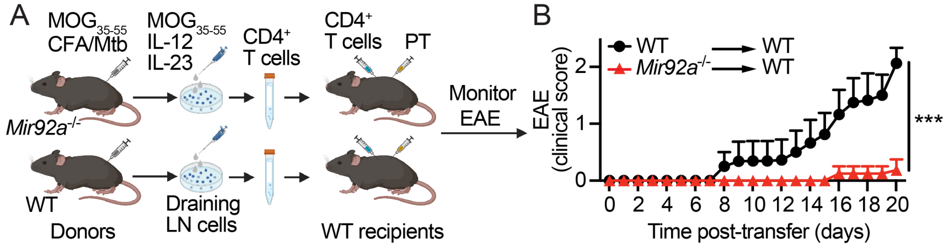

Supplemental Figure 5. T cell-intrinsic miR-92a drives EAE development in an effector $CD4^{+}$ T cell-driven adoptive transfer model of EAE in C57BL/6 recipients.

(A) Adoptive transfer schematic. First, *Mir92a*$^{-/-}$ and WT mice were immunized with $MOG_{35–55}$ and CFA/Mtb. dLNs harvested from these mice were cultured with MOG_{35-55}, IL-23, and IL-12. Finally, effector $CD4^{+}$ T cells were isolated from the dLN cell culture and transferred to recipient naïve WT (C57BL/6) mice. Recipient mice also received PT and were monitored for EAE. Created with BioRender.com. (B) Clinical EAE scores of these WT from A. Data representative of 2 independent experiments. Mean±SEM. ***p<0.001 by Mann-Whitney test (B).

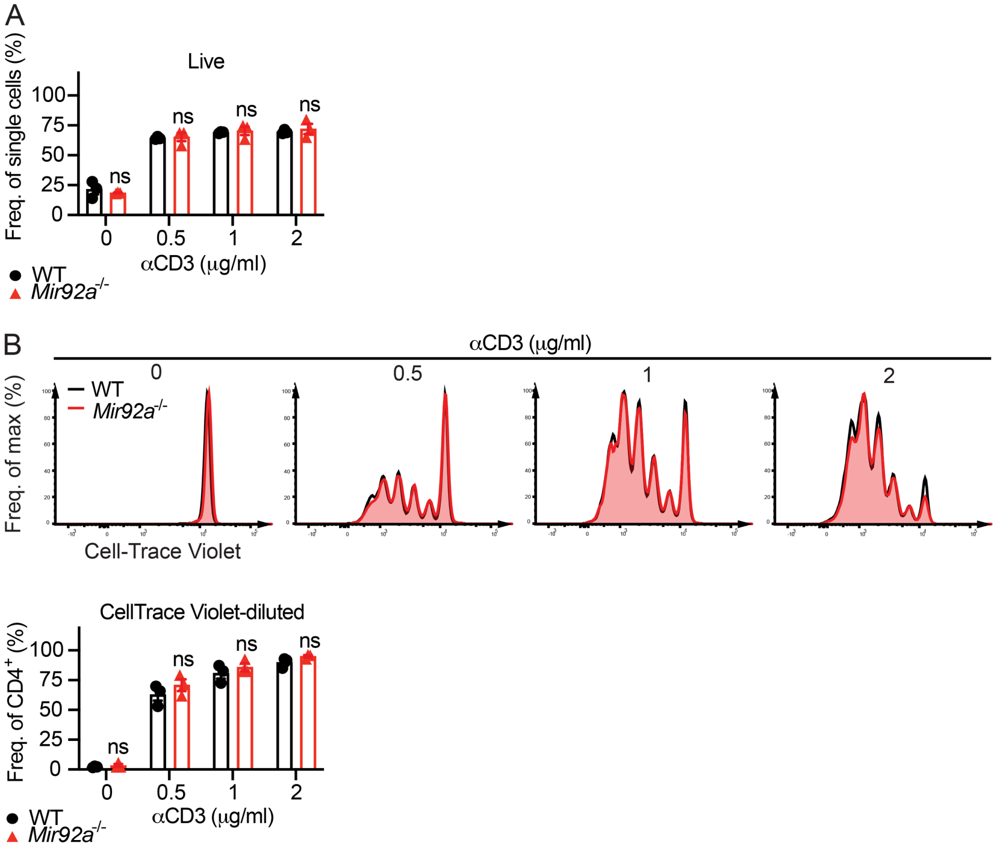

Supplemental Figure 6. MiR-92a deficiency does not alter CD4$^+$ T cell viability and proliferation *in vitro*.

(A) Frequencies of LIVE/DEAD-Aqua$^-$ live populations in WT and *Mir92a*$^{-/-}$ CD4$^+$ T cells stimulated with plate-bound anti-CD3 and anti-CD28 for 4 d. (B) Representative flow cytometric histograms (top) and frequencies (bottom) of CTV-diluted WT and miR-92a$^{-/-}$ CD4$^+$ T cells (n=3). Data representative of ≥2 independent experiments. Mean±SEM. ns=not statistically significant by one-way ANOVA with Sidak's multiple comparison test between WT and *Mir92a*$^{-/-}$ within each condition.

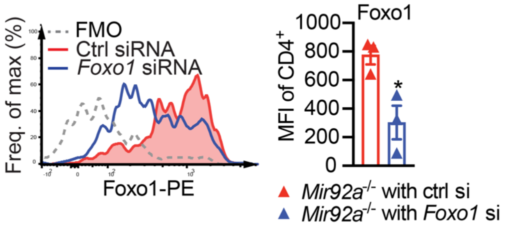

Supplemental Figure 7. *Foxo1* siRNA knockdown.

Representative flow cytometric histograms and MFIs of Foxo1 in naïve *Mir92a*$^{-/-}$ $CD4^+$ T cells transfected with *Foxo1* siRNA, followed by plate-bound anti-CD3 and anti-CD28 stimulation for 72 h (n=3). Data representative of ≥2 independent experiments. Mean±SEM. *$p<0.05$ by unpaired t-test.

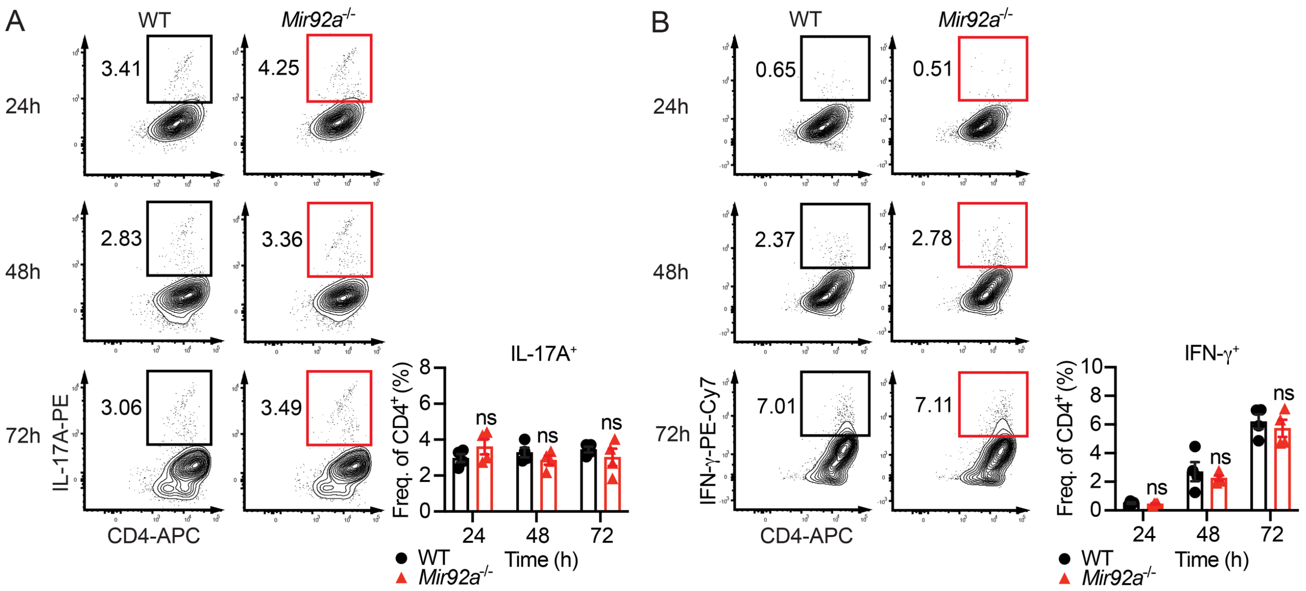

Supplemental Figure 8. *Mir92a*$^{-/-}$ Treg cell phenotypes are unaltered in the absence of inflammatory milieu. (A, B) Naïve CD4^{+} T cells from WT *Foxp3*^{gfp} and *Mir92a*$^{-/-}$*Foxp3*^{gfp} mice were differentiated in vitro into Tregs and sorted for GFP^{+} cells, then cultured with IL-2 for 24, 48, and 72 h (n=4). Representative flow cytometric plots and frequencies of IL-17A^{+} cells (A) and IFN-γ^{+} cells (B) are shown. Data representative of ≥2 independent experiments. Mean±SEM. ns=not statistically significant by one-way ANOVA with Sidak's multiple comparison test between WT and *Mir92a*$^{-/-}$ within each condition.

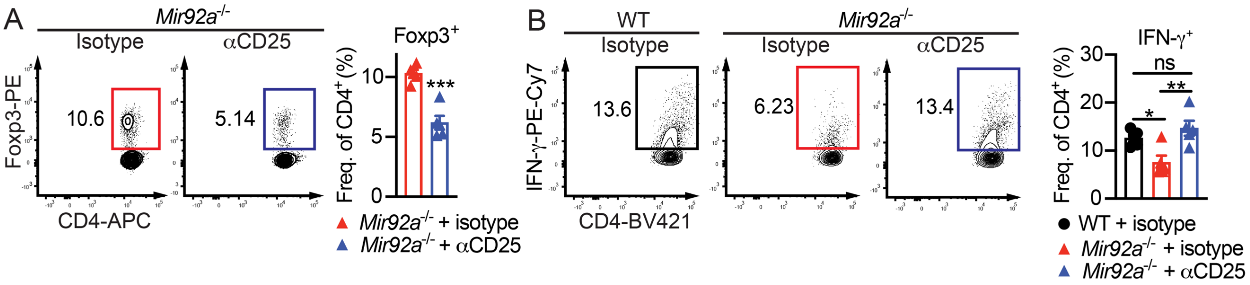

Supplemental Figure 9. Treg depletion in *Mir92a$^{-/-}$* mice restores IFN-γ^{+} Th1 responses during EAE.

(A) Representative flow cytometric plots and frequencies of splenic Foxp3^{+}CD4^{+} T cells at EAE onset from *Mir92a$^{-/-}$* mice treated with either isotype control or anti-CD25 antibody. (B) Representative flow cytometric plots and frequencies of splenic IFN-γ^{+}CD4^{+} T cells at EAE onset from WT and *Mir92a$^{-/-}$* mice treated with either isotype control or anti-CD25. Data representative of 2 independent experiments. Mean±SEM. ns=not statistically significant, *p<0.05, **p<0.01, ***p<0.001 by unpaired t-test (A), or one-way ANOVA with Sidak's multiple comparison test (B).

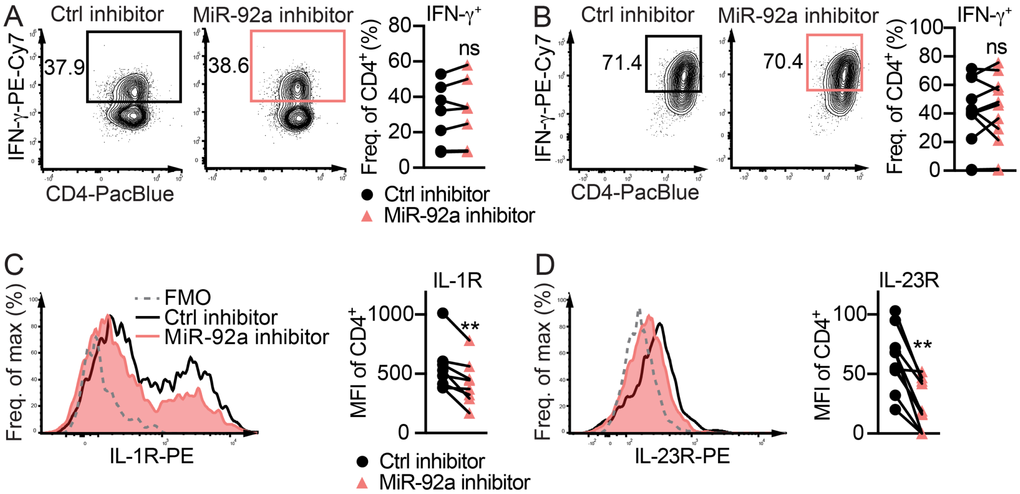

Supplemental Figure 10. MiR-92a inhibitor does not directly alter Th1 induction, but inhibits IL-1R and IL-23R expression under Th17-polarizing conditions in HC and MS patient CD4$^+$ T cells.

(A) Representative flow cytometric plots and frequencies of IFN-γ$^+$ cells in HC naïve CD4$^+$ T cells cultured under Th1-polarizing conditions with control or miR-92a inhibitor (n=7). (B) Representative flow cytometric plots and frequencies of IFN-γ$^+$ cells in MS naïve CD4$^+$ T cells cultured under Th1-polarizing conditions with either inhibitor (n=11). (C, D) Representative flow cytometric histograms and MFIs of IL-1R and IL-23R in MS naïve CD4$^+$ T cells cultured under Th17-polarizing conditions with either inhibitor. MFI values shown are after subtracting the MFI values of FMO controls for IL-1R or IL-23R. Data representative of ≥2 independent experiments. Mean±SEM. ns=not statistically significant, **p<0.01 by Wilcoxon signed rank test.

Table S1. Demographics of healthy control (HC) donors for in vitro T helper cell differentiation assays.

(Relates to Figure 7 and Supplementary Figure 10).

	**HC**
**Number of donors**	23
**Mean age (SD), years**	32 (6.70)
**Female (%)**	69.57
**Male (%)**	30.43
**White (%)**	65.25
**Asian (%)**	30.40
**Black or African American (%)**	4.35
**More than one race (%)**	0
**Unknown or not reported (%)**	0

Abbreviations: HC=healthy control; SD=standard deviation.

Table S2. Demographics of MS patients and HCs for measuring CD4$^+$ T cell miR-92a levels. (Relates to Figure 7J).

	**RRMS**	**HC**
**Number of patients/donors**	28	23
**Mean age (SD), years**	43.02 (12.35)	47.83 (15.06)
**Female (%)**	60.71	69.57
**Male (%)**	39.29	30.43
**White (%)**	86.96	83.33
**Asian (%)**	0	0
**Black or African American (%)**	13.04	8.33
**More than one race (%)**	0	0
**Unknown or not reported (%)**	0	8.34

Abbreviations: RRMS=relapsing-remitting multiple sclerosis; HC=healthy control; SD=standard deviation.

Table S3. Demographics of MS patients for in vitro T helper cell differentiation assays. (Relates to Figure 7 and Supplemental Figure 10).

	**RRMS**
**Number of patients/donors**	16
**Mean age (SD), years**	45.47 (5.99)
**Female (%)**	68.75
**Male (%)**	31.25
**White (%)**	75.00
**Asian (%)**	6.25
**Black or African American (%)**	6.25
**More than one race (%)**	6.25
**Unknown or not reported (%)**	6.25

Abbreviations: RRMS=relapsing-remitting multiple sclerosis; SD=standard deviation.
